# Sustainable Green Nanotechnologies for Innovative Purifications of Water: Synthesis of the Nanoparticles from Renewable Sources

**DOI:** 10.3390/nano12020263

**Published:** 2022-01-14

**Authors:** Szabolcs Bognár, Predrag Putnik, Daniela Šojić Merkulov

**Affiliations:** 1 Department of Chemistry, Biochemistry and Environmental Protection, Faculty of Sciences, University of Novi Sad, Trg Dositeja Obradovića 3, 21000 Novi Sad, Serbia; sabolc.bognar@dh.uns.ac.rs; 2 Department of Food Technology, University North, Trg Dr. Žarka Dolinara 1, 48000 Koprivnica, Croatia

**Keywords:** environmental pollution, water purification, green nanotechnology, heterogeneous photodegradation, TiO_2_/ZnO, plant extract

## Abstract

Polluting the natural water resources is a serious global issue, which is confirmed by the fact that today at least 2 billion people consume water from contaminated sources. The conventional wastewater treatment methods cannot effectively remove the persistent pollutants (e.g., drugs, organic dyes, pesticides) from the aqueous environment. Heterogeneous photocatalysis is a promising and sustainable alternative for water remediation. It is based on the interaction between light irradiation and the semiconductors (e.g., TiO_2_, ZnO) as photocatalysts, but these compounds, unfortunately, have some disadvantages. Hence, great attention has been paid to the nanotechnology as a possible way of improvement. Nanomaterials have extraordinary properties; however, their conventional synthesis is often difficult and requires a significant amount of dangerous chemicals. This concise topical review gives recent updates and trends in development of sustainable and green pathways in the synthesis of nanomaterials, as well as in their application for water remediation. In our review we put emphasis on the eco-friendly, mostly plant extract-based materials. The importance of this topic, including this study as well, is proved by the growing number of publications since 2018. Due to the current serious environmental issues (e.g., global warming, shortage of pure and quality water), it is necessary for the traditional TiO_2_ and ZnO semiconductors to be replaced with the harmless, non-toxic, and more powerful nanocomposites as photocatalysts. Not only because of their higher efficiency as compared to the bulk semiconductors, but also because of the presence of biomolecules that can add up to the pollutant removal efficiency, which has been already confirmed in many researches. However, despite the fact that the application of heterogeneous photocatalysis together with green nanotechnology is absolutely the future in water purification, there are some challenges which have to be overcome. The exact effects of the biomolecules obtained from plants in the synthesis of nanoparticles, as well as in the photocatalytic processes, are not exactly known and require further investigation. Furthermore, heterogeneous photocatalysis is a well-known and commonly examined process; however, its practical use outside the laboratory is expensive and difficult. Thus, it has to be simplified and improved in order to be available for everyone. The aim of our review is to suggest and prove that using these bio-inspired compounds it is possible to reduce human footprint in the nature.

## 1. Introduction

Environmental pollution is an emerging global issue. Even though all of the natural resources are equally exposed to the dangerous effects of different contaminants, the presence of persistent organic pollutants in the aqueous environment is one of the greatest global concerns [1,2]. For instance, according to the UNESCO development report in 2020, 68% of the global population is consuming sanitarily not adequate water and water pollution caused 2.2 billion dollars’ worth of damages to the USA [3].

Over the decades, there were various techniques investigated and used for water purification such as solvent extractions, micro- and ultra- filtrations, sedimentations and gravity separations, flotations, precipitations, adsorptions, etc. [4]. However, the wastewater treatment plants are not able to completely remove the harmful organic pollutants and, accordingly, these compounds are commonly detected in waters within the range of 15–400 ng/L. Furthermore, studies have proved that these persistent substances have negative effects on the living organisms even at concentrations as low as ng/L [1].

To that end, it is necessary to examine and apply other techniques in order to reach the complete mineralization of these pollutants, or at least to turn them into less harmful compounds. Nowadays, great attention is paid to the advanced oxidation processes (AOPs) as possible sustainable techniques for water remediation. The potential water purification strength of AOPs relies on the formation of reactive radicals that are unselective and forceful oxidizing species (*E*^0^ = 2.80 V), which are able to completely mineralize these pollutants to harmless CO_2_, H_2_O and eventual inorganic ions with reaction rates around 10^9^ L/mol s. As with every process, AOPs also have disadvantages such as the presence of scavengers in wastewater, which are in competition with the pollutants for the reactive oxygen species. They can be organic compounds (e.g., humic and/or fulvic acids, proteins, amino acids and carbohydrates) or inorganic ions (like dissolved sulfide, carbonate and bicarbonate, or even bromide and nitrate), depending on the water content [5]. 

**Figure 1:**
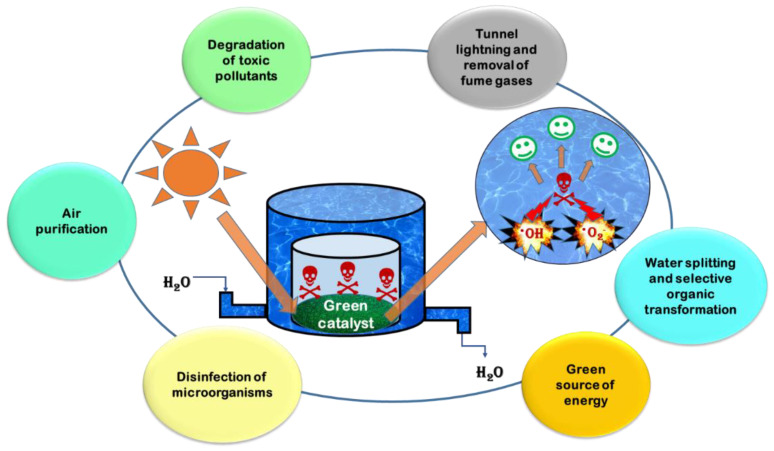
Possible application of heterogeneous photocatalysis.

Among different AOPs, such as the Fenton process, ozonation, catalytic wet peroxide oxidation, catalytic wet air oxidation, electrochemical oxidation or the combination of some of them, photocatalysis, especially heterogeneous photocatalysis is the most actively investigated sustainable process for, but not only, water purification purposes [5]; Figure 1. Heterogeneous photocatalysis is a relatively low-cost, environmentally friendly, sustainable treatment technology. Furthermore, while the chemical and physical processes, such as coagulation, chemical precipitation or adsorption, only transfer the pollutants from one phase to another, photocatalysis together with other AOPs is able to oxidize and destruct a broad range of organic pollutants [6,7]. 

Since the sunlight is a free, renewable and globally available source of energy, photolysis and photocatalysis are promising techniques for the removal of persistent and toxic pollutants from the environment. Both photolysis and photocatalysis are based on the interactions between the electromagnetic radiation and the present compounds; however, in the wastewater treatments mostly the heterogeneous photocatalytic degradation is investigated [8]. This process is based on the application of different semiconductor materials, among which TiO_2_ and ZnO are the most commonly used as photocatalysts. When these materials are exposed to radiation, they can absorb the photons, only if they have higher or equal energy (*hν*) than the semiconductor’s bandgap (*E*g). The result of this process is the formation of electron–hole pairs. In the further steps of photocatalytic degradation, the photogenerated holes can oxidize H_2_O to highly reactive hydroxyl radicals, while on the other hand the electrons reduce dissolved oxygen to superoxide radical anions and later to hydroxyl radicals. These radicals attack the present pollutants that leads to their mineralization, which is desirable (i.e., formation of simple, harmless substances). Unfortunately, heterogeneous photocatalysis has some shortcomings, such as the possible recombination of the photogenerated electron–hole pairs in the absence of adequate electron acceptor in the system [5]. This recombination leads to heat loss and dissipation of heat after their generation in the absence of scavengers [9]. Another drawback of heterogeneous photocatalysis is the high bandgap energy of the commonly used semiconductors, which results that the overlapping between the absorption spectrum of semiconductors and that of the solar radiation reaching the Earth’s surface only occurs in a small fraction, mostly in the UV range [5]. Hence, the use of artificial UV sources is necessary, which are more expensive and UV light requires protective aids for its usage. This issue could be solved by using photocatalysts with bandgap ≤3 eV, which are considered as visible light active photocatalysts [9]. 

**Figure 2:**
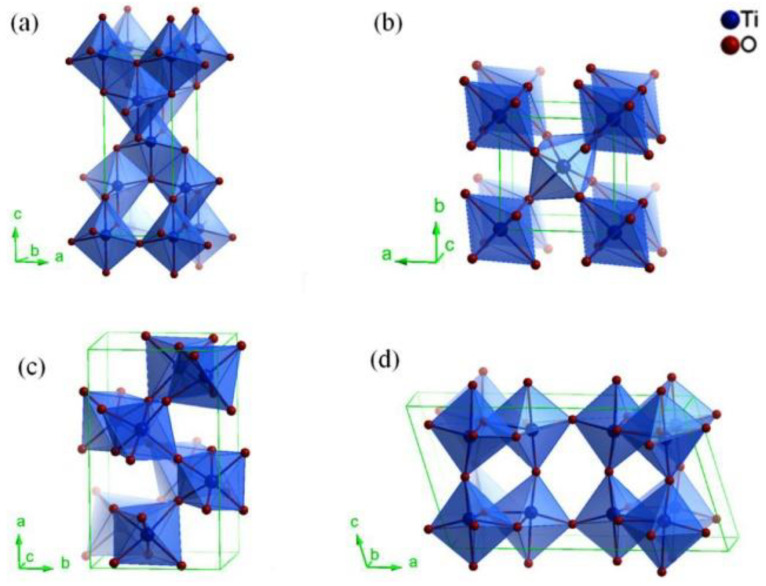
Crystalline structures of TiO_2_ in different phases: (**a**) Anatase, (**b**) rutile, (**c**) brookite, and (**d**) TiO_2_ (B). Reprinted with permission from Ref. [12]. Copyright 2021 ACS publications.

One of the most commonly used and investigated photocatalyst is TiO_2_. The first study about its photoactivity was published in 1932, when TiO_2_ was used for the photocatalytic reduction of AuCl_3_ and AgNO_3_ to Au and Ag. Titanium dioxide possesses different properties which make it suitable for photocatalytic purposes, such as: decent chemical activity, appropriate stability or affordable costs. TiO_2_ is an n-type, wide bandgap semiconductor and has four polymorphs crystalline phases: anatase and rutile (tetragonal), brookite (orthorhombic), and the monoclinic TiO_2_ (B) (Figure 2). All four phases are similar but still different. Namely, all of them consist of TiO_6_ octahedra, but differ in both the distortion of their octahedra units and the manner in which they share edges and corners [10,11].

The most commonly used forms of TiO_2_ are anatase and rutile. The bandgap for anatase is 3.20 eV (corresponding to 384 nm), while in the case of rutile it is 3.02 eV (corresponding to 410 nm) [10]. Some of the basic chemical and physical properties are shown in Table 1.

Even though for years it was believed that TiO_2_ is a nontoxic material and safely can be used for different purposes, in March 2020 the European Food Safety Authority updated the safety assessment about TiO_2_ and declared it as no longer safe as a food additive [14]. Hence, other semiconductors should be also synthesized and examined as potential photocatalysts for water purification. 

ZnO is an eco-friendly semiconductor with wide bandgap energy and safe for daily application because its biocompatibility. ZnO has similar bandgap energy like TiO_2_; however, the ability to absorb a wider range of electromagnetic radiation makes it more attractive in the practical application [8]. Moreover, the quantum efficiency of ZnO powder is also significantly larger than that of TiO_2_ [15]. On the other hand, because of its wide bandgap, its application under natural sunlight (visible region of spectrum) is limited, which results in recombination of photogenerated electron–hole pairs [8]. Taking a look at physical and chemical properties of ZnO it can be seen that this semiconductor has unique characteristics such as chemical stability, electrochemical coupling coefficient, paramagnetic nature and high photostability [16]. On the other hand, longer irradiation could lead to photocorrosion, while in basic medium it comes to self-oxidation as well as to dissolving of ZnO, which also reduces the efficiency of this semiconductor [8,17]. Some of the basic physical and chemical properties of ZnO are shown in Table 2. 

**Figure 3:**
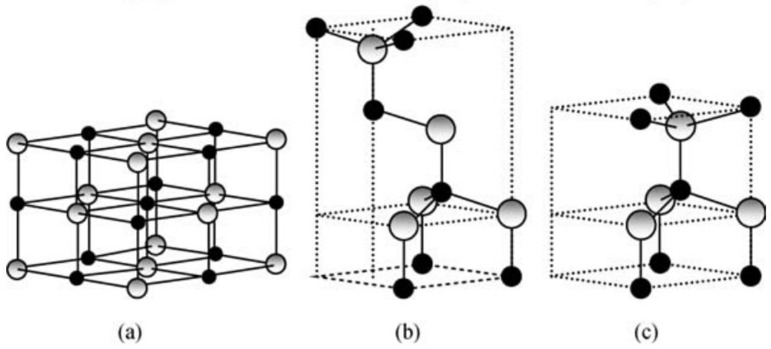
ZnO crystal structures: (**a**) Cubic rock salt, (**b**) cubic zinc blende and (**c**) hexagonal wurtzite. Shaded gray and black spheres denote Zn and O atoms, respectively. Reprinted with permission from Ref. [8], Copyright 2021 Elsevier.

As a part of the II–VI group, ZnO crystallizes in three structures, which are: rock salt, cubic zinc blende and hexagonal wurtzite structure (Figure 3). Under ambient conditions, ZnO is in a thermodynamically stable wurtzite structure, where each anion is surrounded by four cations at the corners of a tetrahedron, and vice versa [19].

**Figure 4:**
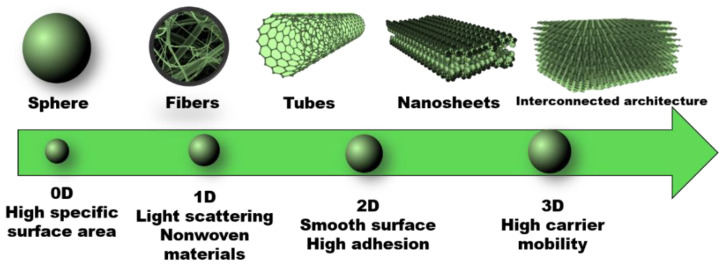
Properties of different nanomaterials form. Adapted from Ref. [22].

As it was mentioned earlier, the two main limitations of the two most commonly used semiconductors as photocatalysts are the wide bandgaps and the rapid recombination of the photogenerated electron–hole pairs [8,10]. Namely, the systems with TiO_2_ and ZnO, because of their wide bandgaps, can successfully utilize nothing but the UV fraction of solar light, which is only about 3−4% of the whole solar spectrum [20]. To this end, it is necessary to enhance their activity under visible light radiation (>400 nm). Various techniques were investigated and applied in order to reduce the bandgap energy and the possible electron–hole recombination of TiO_2_ such as nanostructuring, chemical doping and sensitization [21]. Nowadays, a great attention is paid to the fabrication of different nanomaterials based on TiO_2_ for photocatalytic and other purposes. Nanomaterials can be synthesized in various dimensions as 0D-, 1D-, 2D- and 3D- structures. As an example, spherical TiO_2_ as a 0D-nanomaterial possesses high surface area, which is an important factor in adsorption and photocatalysis. Afterwards, 1D fiber and tube structures can reduce the possible recombination of the photogenerated electron–hole pairs because of the short distance for charge carrier diffusion, light-scattering properties, and fabrication of self-standing nonwoven mats. Furthermore, 2D nanosheets have smooth surface and strong adhesion, while 3D monoliths possess high carrier mobility (Figure 4) [22]. 

Similar approaches were also examined in the case of ZnO. Namely, ZnO as a nanomaterial can also arise in 1D, 2D and 3D nanostructures. For instance, ZnO nanomaterials in 1D group can be in the following forms: needles, nanorods helixes, rings and springs, ribbons, tubes, belts, combs and wires [23]. While in the case of 2D structures ZnO can be obtained such as nanosheet and nanoplate. Furthermore, in 3D group some of the examples for structures of zinc oxide include flower, coniferous, snowflakes, urchin-like, dandelion and other shapes [16]. 

The enhanced photocatalytic activity of the TiO_2_ and ZnO nanomaterials, compared to the bulk form, is the result of their larger surface-to-volume ratio. Moreover, they contain more active sites which also add up to the improved photocatalytic activity [24,25].

On the other hand, the particle agglomeration influences the optical properties of nanomaterials and therefore their ability to absorb and scatter the incoming radiation, also affecting their photocatalytic activity. Furthermore, the agglomeration/aggregation may cause the formation of new surface states at the level of contact between particles, resulting in a change of the photon absorption and of the quantum yield, which is not directly related to the primary properties of the nanomaterials [26]. This phenomena can be successfully avoided by using plant extracts, since the biomolecules can also act as capping or stabilizing agents, so they reduce the possible formation of agglomerates and improve the catalytic activity [27]. 

There are different approaches in the synthesis of nanoparticles (NPs). These synthesis pathways can be separated into three groups: chemical, biological, and physical methods. Chemical techniques can be further divided into solid-phase, liquid-phase and gas-phase processes. Liquid-phase approaches include the following methods: precipitation, coprecipitation method, colloidal methods, sol–gel processing, water–oil microemulsions method, hydrothermal synthesis, solvothermal, and sonochemical, and polyol method. On the other hand, vapor phase approach includes pyrolysis and inert gas condensation methods [16,28]. 

Unfortunately, the application of harmful organic chemicals is necessary for these techniques. Hence, other sustainable pathways should be developed for the synthesis of nanoparticles. At the light of the above mentioned, in this review the reader will be informed about the latest researches, trends and possible alternative green methods in the synthesis and improvement of the photocatalytic activity of TiO_2_ and ZnO.

## 2. Green Synthesis of TiO_2_ and ZnO Nanoparticles Based on Various Plant Extracts

Undoubtedly, there are high hopes for the application of nanomaterials not only as photocatalysts in heterogeneous photodegradation but in other scientific fields too. However, the conventional techniques have their limitations, such as expensive equipment, toxic and non-biodegradable precursors, the need for expertise, low yield of product, as well as long reaction times [29,30].

**Figure 5:**
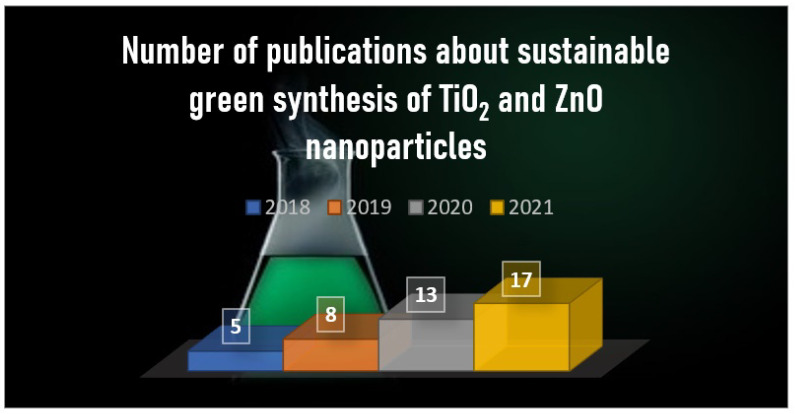
Number of publications on the “green synthesis” topic for photocatalytic purposes (Scopus, September 2021).

To this end, green and sustainable methods should be developed, in order to eliminate or at least decrease the application of harmful chemicals, which negatively affect the environment and living organisms. Fortunately, the scientific society noticed the importance and advantages of green techniques. The number of publications about the topic of green synthesis of different nanocomposites with photocatalytic activity has been increasing continuously since 2018 (Figure 5).

In the following part of this study, some of the latest researches about the possible eco-friendly synthesis of TiO_2_, ZnO, as well as a few special nanomaterials, using different plant extracts instead of harmful organic solutions, will be discussed. The main role of the biomolecules in the synthesis of different NPs is the reduction of metal salts, as well as capping and stabilizing them. Hence, the plant-mediated nanomaterials have a variety of different shapes and sizes comparing to the general, chemical-based synthesis processes. Furthermore, these bio-compounds not only reduce the metal salts, but also functionalize the surface of the newly synthesized NPs, which includes synergistic effects for various applications [31]. Furthermore, the pure plant extracts can also act as catalysts. Namely, these extracts contain biomolecules such as sugars, terpenoids, polyphenols, phenolic acids, alkaloids and proteins. Even though, that these phytochemicals are mainly used as reduction agents, they can also possess photoactivity and can be used as photocatalysts. However, further researches should be conducted in the future to identify these main constituents responsible for this activity, due to the lack of knowledge about these processes [27,32]. The characteristics and efficiency of the green synthesized photoactive nanoparticles are presented in the Table 3.

**Figure 6:**
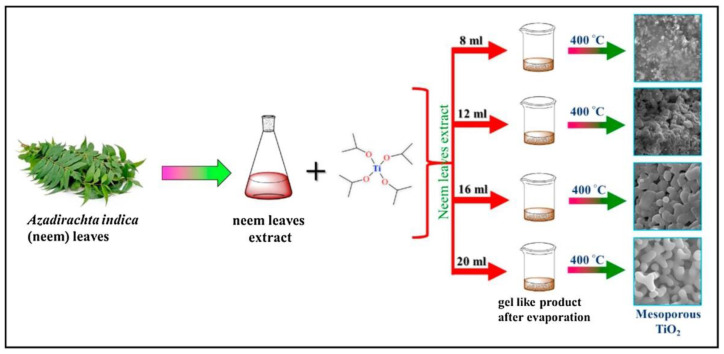
Schematic preparation of TiO_2_ NPs (size range of 240−410 nm) using leaf extract of *Azadirachta indica*. Reprinted with permission from Ref. [29], Copyright 2021 Elsevier.

One example for the green approaches in the synthesis of TiO_2_ nanoparticles is explained in the study by authors Dash et al. [29]. In their work the leaf extract of *Azadirachta indica* was applied. For the preparation of the plant extract, firstly, the fresh, green leaves were collected and washed with tap water. After that, they were also washed using cetyltrimethylammonium bromide (CTAB) solution as well as with distilled water and with 2 M NaOH solution. After washing the leaves, they were dried at ambient temperature. When the drying process was finished, 3 g of the finely cut leaves were mixed with 200 mL distilled water and boiled until the amount of water decreased to 60 mL. Finally, the mixture was filtered and the extract was stored for further use. In order to prepare a mesoporous form of TiO_2_, different amounts of plant extract (8, 12, 16 and 20 mL) were mixed with 0.4 mL of titanium tetraisopropoxide. The reaction mixtures were continuously stirred for 12 h at 35 °C. Then, the temperature was step-by-step increased to 70 °C to eliminate the water from the solution. Finally, after evaporating the water, the obtained product was calcined at 400 °C for 3 h. The formation of white powder proved the successful synthesis of TiO_2_. The newly synthesized NPs, depending on the amount of the plant extract (8, 12, 16 and 20 mL) were named as MTO-8, MTO-12, MTO-16 and MTO-20, respectively (Figure 6).

For the characterization of the green synthesized NPs, various techniques were applied, such as X-ray diffraction (XRD), scanning electron microscopy (SEM), UV-Vis spectrometry, Fourier-transform infrared spectroscopy (FTIR), X-ray photoelectron spectroscopy (XPS) and Brunauer–Emmett–Teller (BET) analysis. The FTIR analysis proved the TiO_2_ structure. All the investigated samples showed characteristic broad peaks in the region of 480–900 cm^−1^, which were corresponding to the stretching vibrations of Ti-O-Ti linkage. Additionally, the findings showed that in the samples with higher amount of leaf extract, the stretching vibration decreased. The SEM images determined that the particle size of the newly synthesized NPs were in the range of 240−410 nm. They also confirmed that the initial amount of the plant extract has an effect on the size of the NPs. Namely, increasing the amount of the extract resulted in decreased particle size, which can be explained with the higher amount of the biomolecules present in the reaction system. The XRD analysis confirmed the anatase phase of TiO_2_ and also showed that the crystallinity of TiO_2_ increased with increasing amount of leaf extract. The average crystal sizes determined by XRD for MTO-8, MTO-12, MTO-16 and MTO-20 were 16.8, 14.5, 13.3 and 12.7 nm, respectively. The UV-Vis results showed absorbance at 335, 322, 304, and 292 nm for the MTO-8, MTO-12, MTO-16 and MTO-20 NPs, respectively. A significant blue shift in the absorbance was observed which is resulted by the higher crystallinity in the samples with higher amount of leaf extract. The bandgaps were also calculated for the newly synthesized NPs, and were 3.08, 2.91, 2.81 and 2.66 eV for MTO-20, MTO-16, MTO-12 and MTO-8, respectively. BET findings showed the surface of MTO-20, MTO-16, MTO-12 and MTO-8 which was found to be 157.35, 91.87, 39.12 and 8.55 m^2^/g. In addition, the pore volumes were also determined (i.e., they were found to be 0.31, 0.26, 0.17 and 0.14 cm^3^/g for MTO-20, MTO-16, MTO-12 and MTO-8, respectively) [29]. The photocatalytic activity of the newly synthesized NPs was also investigated in the removal of Rhodamine 6 G (R6 G) dye under UV irradiation. The obtained results showed that the efficiency of irradiation increased with increasing the time of irradiation. However, the degradation rates of MTO-8 and MTO-12 were significantly slower as compared to the MTO-16 and MTO-20. According to the results it can be seen that 98% of R6 G was degraded by MTO-20 after 57 min of UV irradiation, while in the systems with MTO-16, MTO-12 and MTO-8 the degradation efficiencies were 64%, 27% and 15%, respectively. To sum up, the MTO-20 NPs are promising in the actual water remediation processes; however, these NPs could be more improved since they are efficient in the UV region but not under natural sunlight [29]. 

Besides the above mentioned, Hariharan et al. [33] investigated the possible use of *Aloe vera* in the green synthesis of Ag@TiO_2_ nanoparticles for photocatalytic purposes. The *Aloe vera* gel was prepared by the following: firstly, the leaves were washed with ultrapure water; after that, they were peeled and slit longitudinally, while the gel was squeezed out and collected for further use. Finally, the mixture of 10 mL of *Aloe vera* gel and 100 mL of distilled water was boiled for 2 h at 90 °C. The solution was filtered and stored in refrigerator for further experiments. After the successful preparation of *Aloe vera* gel, the TiO_2_ and Ag@TiO_2_ NPs were also synthesized. Both syntheses were performed using the hydrothermal method. For the synthesis of TiO_2_, 10 mL of titanium (IV) isopropoxide (TTIP) was mixed with 20 mL of distilled water. The solution was heated at 180 °C for 24 h. The homogenous solution was autoclaved and dried at 120 °C for 2 h and the powder was calcined at 500 °C for 5 h. The synthesis of the Ag@TiO_2_ NPs was different. Different concentrations of AgNO_3_ (0.005, 0.010 and 0.015 M), 0.1 M titanium (IV) isopropoxide and 5 mL of *Aloe vera* extract were mixed with 100 mL of water. The mixtures were stirred for 1 h, autoclaved and heated at 180 °C for 24 h. The products were dried at 120 °C for 2 h and calcined at 500 °C for 5 h [33]. The techniques which were used for the characterization are the following: UV-Vis spectrometry, high resolution transmission electron microscopy (HRTEM), energy-dispersive X-ray spectroscopy (EDS or EDX), XPS, XRD and FTIR. The FTIR results showed the presence of *Aloe vera* (i.e., the biomolecules present in the plant). HRTEM analysis proved the differences in the morphology of the pure TiO_2_ and the newly synthesized NPs. The surface of TiO_2_ was very smooth compared to Ag@TiO_2_. Additionally, the concentration of Ag had an effect on the morphology of the NPs. The sizes of the newly synthesized Ag and TiO_2_ NPs were 38 and 57 nm, respectively. XRD analysis proved the anatase phase of the TiO_2_ NPs, while in the case of Ag@TiO_2_ both the anatase and rutile phases were detected. The XDS analysis proved that the Ag has been deposited on the TiO_2_ surface. The UV-Vis spectrometry additionally determined the successful synthesis [33]. Furthermore, the photocatalytic activity of the newly synthesized NPs was also investigated in the photodegradation of picric acid under visible light irradiation. The findings showed that the highest efficiency was reached with 0.010 M Ag@TiO_2_, when after 50 min of irradiation a decent amount of picric acid was removed [33]. 

The green synthesis of a triple nanocomposite was also investigated. Namely, Jiang et al. [34] applied *Cinnamomum camphora* leaf extract in the synthesis of Au-Ag/TiO_2_ catalyst for photocatalytic purposes. The leaf extract was prepared by grinding and washing the leaves, which were followed by their drying at ambient temperature. Two grams of this powder were dissolved in 100 mL of distilled water and stirred for 4 h. Finally, the mixture was filtered and stored at 4 °C for further use. The leaf extract acted as both reducing and capping agent. Whereas the Au-Ag bimetallic and Au-Ag/TiO_2_ NPs were synthesized using the following procedure. First, 0.1 mL of 100 mM AgNO_3_ solution was mixed with 20 mL of leaf extract and stirred at 30 °C for one day. After that, 0.2 mL of 50 mM HAuCl_4_ was added to this solution and the reaction was carried out at 30 °C for 2 h which ended up with the formation of bimetallic NPs. Afterwards, for the synthesis of Au-Ag/TiO_2_ catalyst the previously prepared bimetallic NPs were used. Firstly, 0.5 g TiO_2_ was added to the NPs sol at ambient temperature and stirred for 4 h. This was followed by filtration and drying at 60 °C. Following that, 0.5 g of [BMIM]PF_4_ was mixed with 0.5 g of dried catalyst and 20 mL of deionized water, stirred for 2 h and dried at 60 °C. Finally, the catalysts were calcined at 350 °C as well as at 400 °C for 4 h. For the characterization, different techniques were used: Transmission electron microscopy (TEM), scanning transmission electron microscopy (STEM), XRD, FTIR, inductively coupled plasma with mass spectrometry [34]. The TEM and STEM results confirmed the successful synthesis of the NPs and the presence of Ag and Au alloyed metals. Additionally, the TEM analysis determined a spherical shape and a uniform size of 12.6 ± 1.7 nm. The FTIR spectra proved the successful green synthesis of these NPs, as well as the oxidants and capping agent ability of the *Cinnamomum camphora* leaf extract. The XRD analysis proved the anatase phase of TiO_2_ without any obvious changes when Au-Ag metals were added. The photocatalytic activity of the green synthesized NPs was also investigated in the photodegradation of methyl orange (MO) under UV-Vis irradiation. Findings showed a high activity of the newly synthesized NPs. Specifically, with the fresh Au-Ag/TiO_2_ 89.4% of MO was degraded after 60 min of irradiation. In addition, the photocatalytic activity was also examined in the case of mix dyes (MO; methylene blue, MB; and rhodamine B). The results showed that all of the absorption peaks in the UV-Vis spectra disappeared after 60 min of irradiation, which confirmed the high photodegradation efficiency. In addition, the stability of the NPs was also questioned. According to the results it was concluded that after 5 cycles of photodegradation, there were no changes in the activity of the NPs, which confirmed a possible industrial utilization usage of these NPs [34].

In the work by Rufai et al. [35], the synthesis of TiO_2_ was examined using *Deinbollia pinnata* leaves for photocatalytic purposes. The plant extracts were prepared using sonication method. The leaves were put into several conical flasks and mixed with 150 mL of organic solvent (*n*-hexane, ethyl acetate and methanol) in a sonicator for 10 min with agitation. After that, the samples were left under ambient conditions for 24 h. Finally, they were concentrated at 40 °C using a rotary evaporator. For the preparation of the TiO_2_ NPs the sol–gel method was used with TTIP as the precursor. Volume of 4 mL of TTIP was mixed with 20 mL of deionized water and stirred for 30 min at 30 °C. After that, 0.2 g/mL of plant extract was dropwise added and stirred for 4 h at 50 °C. This was followed by keeping the reaction mixture at ambient conditions during 48 h for aging process. The obtained suspension was rinsed with ethanol and centrifuged at 10,000 rpm for 30 min. Finally, the samples were dried (95 °C for 24 h) and calcined at 500 °C for 5 h. For the characterization different techniques were used: XRD, SEM, EDX, UV-Vis spectrometry, BET, and FTIR [35]. The XRD results confirmed the anatase phase of TiO_2_ NPs in great purity with average crystal size in the range of 19–21 nm. Additionally, the XRD patterns showed that the used organic solvent had effects on the synthesized products. More precisely, a significant crystallinity and peak sharpness of green TiO_2_ with ethyl acetate applied for extraction were observed, as compared to those of methanol and *n*-hexane. The SEM images confirmed that TiO_2_ NPs were successfully produced in nano-sized. EDX analysis proved the presence of Ti and O in the newly synthesized NPs with mass ratios of 61% and 35%, respectively. The UV-Vis spectrometry showed that the wavelength dependent absorbance was within the range of 300–500 nm. The bandgap energy was also calculated and found to be 3.2 eV, which is typical for the TiO_2_ anatase phase. In addition, according to the BET analysis it can be seen that the surface of the newly synthesized green NP was 31.77 m^2^/g. The FTIR analysis additionally proved the successful synthesis, since there was a peak at 537 cm^−1^ which represented a characteristic peak of Ti-O-Ti vibration. The photocatalytic activity of the newly synthesized NPs were investigated in the degradation of MO dye under 150 min of UV irradiation. The obtained results showed that 97.53% of MO was degraded after 150 min of UV irradiation. These data indicate that a high performance can be reached within a short time thanks to the enhanced charge carrier’s separation and increased surface area [35]. 

Udayabhanu et al. [36] investigated the possible use of *Euphorbia hirta* leaf extract in the synthesis of TiO_2_ nanoparticles for photocatalytic application. For the extract preparation the leaves were firstly washed from impurities and the extract was prepared using microwave irradiation. Mass of 20 g of the leaves were cut into small pieces, homogenized and mixed with distilled water. The mixture was boiled under microwave irradiation for 10 min. The extract was cooled down to room temperature and filtrated. The obtained *Euphorbia hirta* extract acted as both reducing and capping agent for TiO_2_ synthesis. For the green synthesis, a mixture of 100 mL of leaf extract and 900 mL of 5 mM TiO_4_ solution was prepared and incubated under sunlight for 24 h. To complete the formation of NPs, the reaction mixture was centrifuged at 8000 rpm for 10 min and the formed pellet was dissolved using several drops of hydrofluoric acid (40%) and washed several times. Finally, it was dried at ambient temperature for two days to turn it into powder form. For the characterization the following techniques were used: UV-Vis spectrometry, FTIR, SEM, EDX and XRD [36]. The UV-Vis spectrophotometry was used to confirm the reduction of titanium ions. Peak has appeared in the spectra at 420 nm, which was originated from the TiO_2_ in the examined samples. The FTIR analysis determined the presence of biologically active compounds that were adsorbed to the surface of green synthesized NPs. It is believed that the bioactive substances may have involved in the bio-reduction of TiO_2_ NPs. The XRD patterns confirmed the cubic phase of titanium. Furthermore, the SEM images showed that the new NPs had a spherical shape with sizes between 20 and 50 nm. In addition, the EDX analysis showed intensive signal from titanium ions and weak signals of C, K and O. The weak signals can be explained with the presence of the biomolecules. Experiments of photocatalytic degradation were also conducted in order to investigate the possible photoactivity of the green synthesized NPs in the degradation of methylene blue (MB), MO, alizarin red (AR) and crystal violet (CV) under 6 h of direct sunlight irradiation. The obtained results showed that the highest removal efficiency was reached in the case of MB where 95.7% of it was removed, while in the systems with CV 86.8%, AR 81.3%, and with MO 77.5% was removed, respectively, after 6 h of irradiation [36]. 

**Figure 7:**
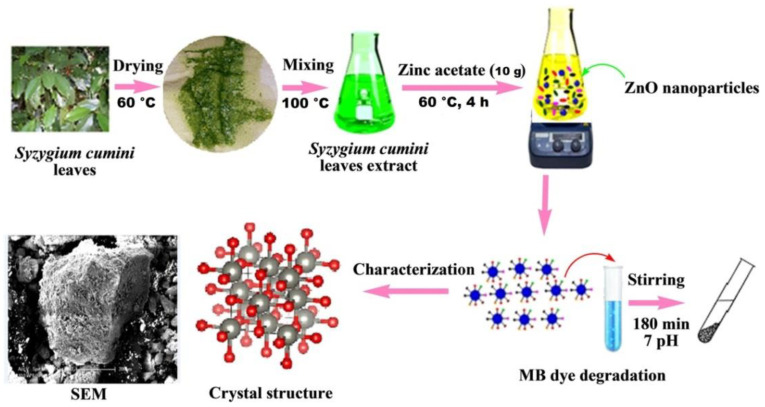
Preparation scheme of ZnO nanoparticles. Reprinted with permission from Ref. [37], Copyright 2021 Elsevier.

Sadiq et al. [37] investigated the green synthesis of ZnO using *Syzygium Cumini* leaves extracts and its photocatalytic efficiency. For washing and preparing the extracts double-distilled water was used. After that, extracts were dried in an oven at about 60 °C, and after 30 min the dried leaves were taken out of the oven and kept at room temperature. One hundred grams of these leaves were dissolved in 800 mL deionized water and boiled with continuous stirring at about 100 °C for 35 min. Finally, the mixture was filtered to obtain a clear solution and stored in refrigerator at 4 °C. After that, there were two approaches to finish the synthesis, using or water or ethanol as a solute and zinc acetate as a precursor in both cases (Figure 7).

Summarizing the results, it can be concluded that ZnO nanoparticles with anisotropy and crystallized morphology were successfully synthesized. Results of the XRD analysis indicated that ZnO were in a well-crystallized hexagonal wurtzite structure with size of 11.35 nm. The crystallized pattern also reflected that the sample was without any impurity and was well crystallized. SEM analysis was also made, which showed that ZnO nanoparticles had agglomerated structural morphology with size around 200 μm. Investigating the photocatalytic efficiency of newly synthesized ZnO, it was found that the ZnO NPs possesses very good photocatalytic activity against molecules of dye. Namely, 91.4% of MB was removed after 180 min of sunlight irradiation, which indicates the possible application of these NPs in wastewater treatment plants [37]. 

There are also studies in which the synthesis of ZnO was based on pullulan [38,39]. In this case the precipitation method was applied with 5 g of pullulan dissolved in appropriate amount of water. As a precursor, Zn(NO_3_)_2_·6H_2_O was used and in the end the precipitate was centrifuged, washed with water and dried overnight in an oven. Finally, the obtained powder was calcined at different temperatures, namely at 400 [38] and 500 °C [39]. In order to investigate the structural properties, XRD analysis was conducted. There were no additional peaks present in the XRD patterns, which indicated that the prepared ZnO particles were free of impurities, in both cases. Besides that, the optical properties of newly synthesized ZnO were also investigated. When the ZnO nanoparticles were calcined at 400 °C, a sharp absorbance peak of ZnO NPs was obtained within the 366–371 nm range. With increasing zinc salt’s amount, the maximum absorbance shifted to a higher wavelength (red-shift). This might be a reason of the increased particle size. On the other hand, at 500 °C in the case of zinc oxide microflowers (ZnO-MFs) the absorbance reduced at wavelength above 370 nm and this can be assigned to the direct bandgap of ZnO-MFs. In addition, morphological studies were also conducted. Based on the TEM analysis, the average particle size of ZnO NPs without pullulan was significantly large, 110.86 nm, while the smallest ZnO NPs were obtained using 5 g of zinc precursor with an average particle size of 58.13 nm. Furthermore, the morphology of ZnO-MFs was investigated using field emission scanning electron microscope (FESEM). It was observed that the particles exhibited flower-like shape. As the pullulan amount increased, the ZnO-MFs particles become smaller and pores were generated. Furthermore, the photocatalytic efficiency of both ZnO particles was also investigated in the removal of MO under UV irradiation. According to the obtained results it can be seen that 97% of MO was degraded after 300 min of irradiation, hence these nanomaterials can be efficiently used in the removal of drugs or organic dyes from aqueous environment [38,39].

Narath et al. [40] investigated the *Cinnamomum tamala* leaf extract in the synthesis and stabilization of ZnO NPs. Firstly, the leaves were dried and ground in order to get a fine powder. After that, 10 g of this powder was mixed with 250 mL of distilled water and stirred at 80 °C for 1 h. Finally, the solution was filtrated and stored in refrigerator for further use. In the synthesis, as a precursor for Zn the Zn(NO_3_)_2_·6H_2_O metal solution was used. For the characterization there were used different techniques: XRD analysis, FTIR, UV-Vis analysis, scanning electron microscope-energy-dispersive X-ray spectroscopy (SEM-EDX) analysis, TEM, Raman spectroscopy and XPS. Furthermore, photocatalytic studies of green synthesized ZnO NPs in the degradation of MB were also carried out [40]. According to the obtained results it can be concluded that the newly synthesized ZnO NPs were hexagonal crystalline in nature and had an average size of 35 nm. The bandgap obtained from the Tauc plot was 3.24 eV. In addition, considering the results of UV-Vis spectrometry it can be seen that there was a small number of weak intense sharp peaks between 250–350 nm, which indicates the surface defects due to the recombination of electrons in the conduction band and holes in the valence band, which also specify the monodisperse nature of ZnO NPs. According to the photocatalytic experiments it can be concluded that the green synthesized ZnO NPs have outstanding efficiency in the degradation of organic dye. Namely, according to the photocatalytic experiments it can be seen that 98.07% of MB was degraded after 90 min of direct sunlight. Thus, this sustainable method provides a better alternative over other conventional methods of ZnO synthesis [40]. 

Park et al. [41] have investigated the application of *Gynostemma pentaphyllum* extracts in the synthesis of ZnO and its efficiency in the photocatalytic degradation of malachite green dye. The preparation method of the leaf extract was the following: washing the samples and grinding them into a fine powder; autoclaving them for 40 min at 100 °C to get extract without any contamination; after that filtering and centrifuging the autoclaved extract at 4500 rpm at room temperature for 15 min. Finally, collecting the supernatant which was then stored in the refrigerator for further use. In the synthesis of ZnO the co-precipitation method was applied. The leaf extract acted as a reduction agent, while zinc nitrate salt and NaOH worked as precursors. A total of 20 mL of the 5% (*w*/*v*) extract was mixed with 80 mL of distilled water, and 0.1 mM of zinc nitrate salt was combined with this solution and constantly stirred until achieving the homogenous mixture. After that, the temperature was set to 70 °C and 0.2 M aqueous solution of sodium hydroxide was slowly added. Finally, the mixture was stirred at 500 rpm for 2 h and after 12 h of settling down the supernatant was removed. For the characterization the following techniques were used: UV-Vis spectrometry, field emission transmission electron microscopy (FE-TEM), selected area (electron) diffraction (SAED), EDX, XPS and XRD. Furthermore, the photocatalytic activity of the newly synthesized ZnO NPs was also investigated under UV lamp in the removal of malachite green dye (MG) (10 mg/L) [41]. According to the XRD analysis it can be concluded that the crystalline core size was 35.41 nm, while the FE-TEM analysis showed that the newly synthesized NPs had hexagonal shape. Taking into account the XPS results it can be seen that NPs were pure and there were not found any impurities. The photocatalytic studies showed that, after 180 min of UV irradiation, 89% of MG was removed. Furthermore, the observations showed these ZnO nanoparticles could be used for more than five cycles with a slight reduction in their activity [41]. 

Abdullah et al. [42] made a comparative study of chemically and bio-inspired synthesized ZnO nanoparticles for photocatalytic degradation of organic pollutants in wastewater treatments. The preparation of the aqueous extract of *Cavendish bananas* was done in the following steps: First, the banana flesh was removed; secondly, the peels were cleaned to get rid of any foreign materials. After that, the peels were dried at ambient temperature and 100 g of small-cut pieces of peels were added to 100 mL deionized water; and the mixture was boiled for 20 min at 80 °C. Finally, the mixture was filtered twice, using Whatman No. 1 filter papers. For the green synthesis of ZnO nanoparticles 1 mL of the prepared extract was mixed with 50 mL of 0.02 M zinc acetate dihydrate (Zn(CH_3_COO)_2_·2H_2_O) solution and stirred for 10 min. Afterwards, the pH of the mixture was adjusted to 12 by adding 2 M NaOH solution. The mixture was left to continuously stir for 3 h at room temperature. Then, the suspension was centrifuged at 3500 rpm for 10 min. Finally, the precipitate was washed with deionized water and in the end dried in an oven overnight at 60 °C. For the chemical synthesis, firstly, 100 mL of Zn(CH_3_COO)_2_·2H_2_O (0.25 M) was added to 100 mL of 1 M NaOH at ambient temperature with stirring continuously for 1 h. After that, the precipitate was collected and rinsed with deionized water and absolute ethanol prior to drying in an oven for 5 h at 100 °C. In the end, the dried precipitate was calcined at 250 °C for 3 h. For the characterization, the following methods were used: XRD, SEM with EDX, FTIR, TEM, UV-Vis spectrometry, dynamic light scattering (DLS) and XPS. In addition, photocatalytic experiments were also carried out in order to investigate the efficiency of newly synthesized ZnO NPs in the removal of hazardous organic contaminants (organic dye) under xenon lamp equipped with UV filter [42]. Based on the XRD results it can be concluded that the biosynthesized ZnO was crystallized with hexagonal wurtzite structure and the size of 15.3 nm without undergoing calcination process. Results of FTIR analysis indicated the presence of biological components in the newly synthesized catalyst. According to the SEM and TEM analysis, it can be seen that the ZnO particles were uniformly distributed. The photoluminescence and UV–DRS studies showed a red emission in the visible region and indirect band gap of 3.18 eV. Additionally, the isoelectric point of ZnO NPs was found at pH 7.2. On the other hand, the green ZnO had superior photodegradation efficiency and reusability compared to the chemically synthesized ZnO. The photocatalytic investigation was conducted on three organic dyes: Basic Blue (BB9), CV and Congo red (CR) using simulated sunlight. According to the obtained results it can be concluded that in the case of BB9 the complete degradation was reached after 90 min of irradiation. On the other hand, for the cases of CV and CR, 99.79% and 81.70% were removed after 420 min of irradiation, respectively. The main reason is probably the ZnO NPs smaller sizes, larger surface area, and the presence of biological components in them [42]. 

Ekennia et al. [43] investigated the application of the leaf extracts of *Alchornea laxiflora* in the synthesis of ZnO and its application in the photocatalytic degradation of CR organic dye. For the aqueous extract the *Alchornea laxiflora* leaves were dried and ground into fine powder in a mortar. Mass of 4 g was dissolved in 200 mL of deionized water and was heated at 80 °C for 45 min. After that, the extract was filtered using Whatman filter paper and stored in the refrigerator for further use. For the sustainable synthesis of ZnO 2 mL of the leaf extract were added in drops to the ZnCl_2_ salt solution. The pH of the mixture was set to 11 using 0.2 M NaOH solution. After that, the mixture was stirred for 2 h and filtered under gravity. The product was washed for several times and dried in the oven at 60 °C for 48 h. The same procedure was repeated using 1, 1.5 and 3.5 mL of the plant extract [43]. The tyrosine inhibitory activity was also investigated using spectrophotometric method which is in detail discussed in the works Basavegowda et al. [44] and Basavegowda et al. [45]. In the photocatalytic experiments, 8 mg of newly synthesized catalyst was added to the solution of 1.5 mg/L of CR dye. According to the results of optical analysis and to the Tauc’s plot it can be seen that the bandgap energy was depending on the volume of used plant extract, namely 3.67 eV (1 mL plant extract), 2.56 eV (1.5 mL plant extract), 2.50 eV (2 mL plant extract), and 2.89 eV (3.5 mL plant extract). FTIR spectroscopy confirmed the presence of natural compounds (e.g., flavonoids, tannins and alkaloids) in the newly synthesized ZnO NPs. The SEM results showed that the predominant shape is quasi-hexagonal with increased size of ZnO from 29 to 38 nm when the volume of the plant extract was varied from 1 to 2 mL. Photocatalytic experiments showed that the green synthesized ZnO nanoparticles had high efficiency in the degradation of CR organic dye in a very short duration time (i.e., 87% of CR was removed after 60 min of direct solar irradiation). According to these findings, it can be expected that these NPs could be used in industrial wastewater treatments as well as nontoxic bioactive molecules due to its tyrosinase inhibitory potentials [43]. 

The possibilities of the banana peels extract in the synthesis of ZnO were also investigated by Fernanda et al. [46]. For the preparation of the extract, 150 g of banana peel was washed and boiled in 150 mL deionized water. Then, the boiled peels were pounded until smooth and mixed with the earlier used water. After that, the mixture was filtered using a cloth. For the synthesis of ZnO, 500 mL of ZnSO_4_ (0.0783 M) was added to 129.47 mL of banana extract. The pH was set to 12 by adding NaOH solution and the mixture was washed using a Buchner funnel until the filtrate showed neutral pH. The product after the filtration was dried in an oven at 60 °C until its weight was constant. For the characterization, XRD analysis was used. The findings showed that the size of crystals was 18.86–20.72 nm which depends on the used type of banana (i.e., on the concentration of secondary metabolites contained in the extracts). The synthesized particles are believed to be useful photocatalysts in water purification [46].

Studies were conducted in order to investigate the possibility of jujube fruit in the green synthesis of ZnO. Golmohammadi et al. [47], for the preparation of the fruit extract, first washed and dried the fruits. Secondly, they were ground by a mechanical grinder. Afterwards, 20 g of this powder was refluxed at 80 °C for 30 min. Finally, the aqueous extract of the jujube fruits was filtered and stored for further use. For the green synthesis, 25 mL of Zn(NO_3_)_2_·6H_2_O (0.05 M) and 100 mL of fruit extract were stirred at ambient temperature for 30 min. The color of the mixture changed after 1 h, which indicated the formation of colloidal ZnO NPs. Accordingly, the solution was continuously stirred for another 4 h at 80 °C. After that, the solution was cooled and centrifuged at 6000 rpm. The precipitates were separated, washed several times and dried at room temperature. Finally, the NPs were ground in an agate mortar and placed in a furnace at 500 °C for 3 h to remove any unreacted chemical or other contaminants. For the characterization of the newly synthesized NPs the following techniques were used: FTIR, XRD, TEM, SEM, EDX and UV-Vis absorption spectrometry [47]. Furthermore, experiments were also conducted in order to investigate the photocatalytic efficiency of the green synthesized ZnO NPs in the degradation of MB and eriochrome black-T organic dyes. Findings showed that the newly synthesized NPs had hexagonal wurtzite structure with uniform particle size of 19 nm. The bandgap energy of the ZnO NPs was also determined using Tauc plot, and it was 3.05 eV. According to the kinetics of the photocatalytic degradation it can be seen that the NPs successfully removed 85% of both dyes from the wastewater after 300 min of direct solar irradiation [47]. 

*Prunus cerasifera* leaves were also examined in the synthesis of ZnO nanoparticles by authors Jaffri and Ahmad [48]. Firstly, the plant leaves were cut into small pieces in order to get a fine powder. For the extraction 10 g of prepared powder was mixed with 100 mL of DI at 60 °C for 20 min. Then, the mixture was centrifuged at 5500 rpm for 10 min which is followed by double filtration. Finally, the extract was stored in the refrigerator for further use. For the ZnO synthesis, firstly 20 mL of the extract was heated at 60–80 °C with continuous stirring. Twenty minutes later 3 g of Zn(NO_3_)_2_ was added and the mixture was heated and stirred in whole time. In order to get white ZnO NPs from yellow suspension, the pH was adjusted at 8. After that, the suspension was centrifuged at 6000 rpm and washed with ethanol. Precipitates were separated and dried at 120 °C in oven for 2 h. Finally, the product was calcined at 200, 400 and 600 °C, and ground to fine powder in a mortar. Characterization made using different techniques: UV-Vis spectrometry, FTIR and XRD. Photodegradation experiments were also carried out to investigate the efficiency of newly synthesized ZnO NPs for the removal of different organic dyes (bromocresol green, BG; bromophenol blue, BB; methyl red, MR; and MB) [48]. According to the results obtained by UV-Vis spectroscopy and FTIR, it can be seen that the ZnO NPs were successfully synthesized using this method. XRD analysis proved the phase purity of the nanoparticles with an average crystallite size of 12 nm. Based on SEM data the findings showed that the NPs were in the nanoscale with spheroidal morphology. Furthermore, the photocatalytic experiments showed that the newly synthesized NPs had an appropriate efficiency. Namely, 93.12% of BG, 90.54% of BB, 88.49% of MR and 76.76% of MB were degraded after 10–12 min of direct solar irradiation [48]. 

In the work of Kahsay [49] the possible application of *Becium grandiflorum* extract was investigated for antimicrobial activity and removal of MB. Firstly, the plant extract was prepared as follows: the leaves were washed and dried at ambient temperature for 14 days; then the dried leaves were crushed into powder; finally, in order to prepare 1% plant extract, 1 g of this powder was dissolved in 100 mL deionized water and heated at 40 °C for 20 min. The solution was filtered and stored for further use. In the synthesis of ZnO, Zn(CH_3_COO)_2_·2H_2_O was used as precursor, while leaf extract of *Becium grandiflorum* as reducing and capping agent. Best products were obtained under the following conditions: 1 M solution of Zn(CH_3_COO)_2_·2H_2_O; pH 9; 1% leaf extract; at 60 °C for 30 min. The appearance of yellow color was the proof of successfully formed ZnO NPs. For the characterization of the newly synthesized ZnO NPs different techniques were used, such as: UV-Vis spectrometry, FTIR, XRD, SEM and EDS. The UV-Vis spectrometry results proved the presence of ZnO NPs [49]. The FTIR spectra showed characteristic peaks for *Becium grandiflorum* which indicated that the leaf extracts were successfully applied as reducing and capping agents. XRD results showed that the ZnO NPs had hexagonal wurtzite structure with average crystal size of 20 nm. Furthermore, the EDS analysis showed that the percent composition of Zn and O in ZnO NPs were 40.38% and 59.62%, respectively. The photocatalytic efficiency of the newly synthesized NPs was also investigated in the removal of MB under UV irradiation. It was found that 69% of MB was degraded after 200 min of irradiation. Furthermore, adsorption studies were also conducted on the ZnO NPs. The findings showed that 71.53% of MB was removed when the adsorbent mass and dye concentration were 25 mg and 25 mg/L, respectively, after 180 min of treatment [49]. 

**Figure 8:**
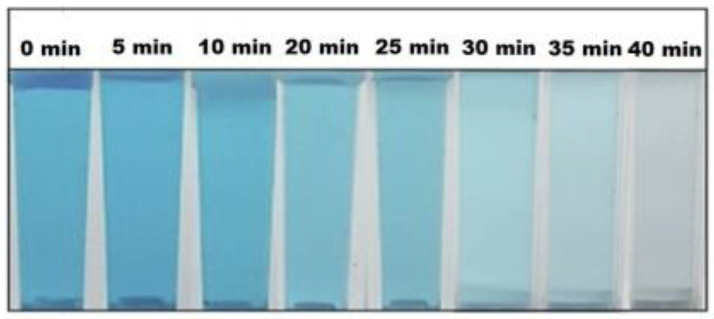
Photodegradation of MB using *Codonopsis lanceolata*-mediated ZnO NPs under UV irradiation. Reprinted with permission from Ref. [50], Copyright 2021 Elsevier.

Lu et al. [50] examined the application of *Codonopsis lanceolata* root extract in the synthesis of ZnO NPs and also investigated its photocatalytic activity in the degradation of MB. In the synthesis of ZnO, firstly the extract was prepared. The roots were washed and cut into pieces and dried overnight at 40 °C. Then, 20 g of the roots were autoclaved with 100 mL of distilled water for 20 min at 60 °C. After cooling, the mixture was filtered through Whatman No. 1 filter and centrifuged at 10,000 rpm. Finally, the separated pellet was washed with water and ethanol, and dried in a vacuum oven at 60 °C which resulted the oxidation of Zn(OH)_2_ to ZnO white powder. For the characterization various techniques were used: UV-Vis spectrometry, TEM, SAED, EDX and XRD [50]. The presence of ZnO NPs was proved by the UV-Vis spectra, where a peak was observed at 356 nm, which is the characteristic band for zinc oxide. SAED results showed slight difference in the thickness of the newly synthesized NPs, while the TEM analysis determined a flower-like structure with the size of 500 nm. The EDX findings showed the atomic percentage of ZnO NPs, which was 47.08% of zinc and 52.92% of oxygen. Furthermore, XRD analysis showed a hexagonal wurtzite form of ZnO NPs. FTIR spectra confirmed the presence of *Codonopsis lanceolata* which proves the successful green synthesis of ZnO. The photocatalytic activity of the newly synthesized ZnO (1 mg/mL) was also examined in the degradation of MB under UV irradiation at ambient temperature. According to the findings, it can be seen that 90.3% of MB was removed after 40 min of irradiation (Figure 8) [50].

*Peltophorum pterocarpum* leaf extract was also examined for the sustainable synthesis of ZnO NPs [51]. The leaves were freshly collected and washed with water. After drying, leaves and water were taken at a weight ratio of 1:10 and boiled for 1 h. After that, the mixture was filtrated and store in cold for further use. In the synthesis of ZnO NPs, 0.05 M zinc acetate dehydrate was used as a precursor. Additionally, 200 mL of leaf extract and 200 mL of 1 M NaOH solution was dropwise added to the solution, until a pale white precipitate was obtained. For the characterization of the newly synthesized NPs, different techniques were used such as UV-Vis spectrometry, FE-SEM, EDS, XRD, thermal gravimetric analysis (TGA) and FTIR [51]. During the spectral analysis, a peak appeared in the UV region at 365 nm. The appearance of peaks between 300 and 400 nm proved the successful synthesis of ZnO. The SEM image showed a flower-shaped ZnO nanostructure, while the EDS analysis confirmed the presence of zinc and oxygen. The percentages of zinc and oxygen in the synthesized nanoparticles were 73.65% and 17.41%, respectively. The XRD results confirmed the standard hexagonal wurtzite structure with the size of 11.64 nm. The TGA findings showed a significant weight loss with increasing temperature from 27 °C to 800 °C. The FTIR spectra of the ZnO NPs confirmed the presence of various functional groups. The photocatalytic activity of the newly synthesized ZnO NPs was also investigated. The degradation experiments were conducted on MB under natural sunlight irradiation. According to the results, it can be seen that almost 95% of MB was degraded after 120 min of irradiation [51].

**Figure 9:**
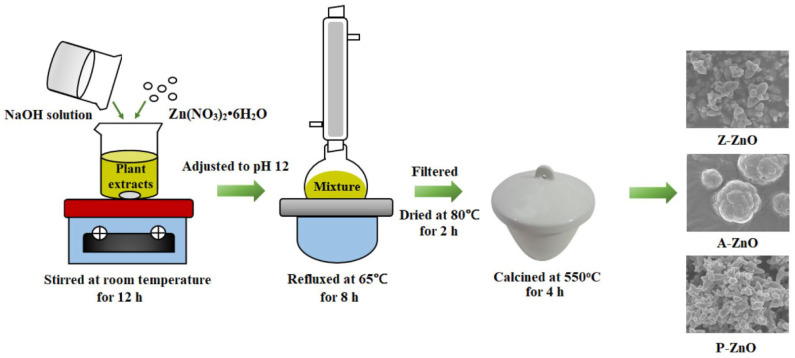
Synthesis of ZnO NPs, using three different plant extracts. Reprinted with permission from Ref. [52], Copyright 2021 Springer.

Quek et al. [52] investigated the application of *Zea mays* husk, *Artocarpus heterophyllus* peel and *Punica granatum* peel extracts in the synthesis of ZnO NPs for photocatalytic purposes. Firstly, the collected plant by-products were washed and put into an oven at 40 °C for two days, in order to remove all the moisture from the samples. After that, they were milled and each of them was extracted with Soxhlet apparatus using 50% ethanol solvent for 2 h and dried over a water bath at 90 °C for 2 h. Finally, the extracts were filtered and stored in refrigerator for further use. For the synthesis of the ZnO nanoparticles with three different plant extracts, the co-precipitation method was applied under low-temperature reflux conditions. A total of 2.97 g of the precursor (Zn(NO_3_)_2_·6H_2_O) was dissolved in 100 mL of *Punica granatum* peel extracts, and the necessary basic environment (pH 12) was ensured using NaOH aqueous solution. After stirring for 12 h, the obtained mixtures were refluxed at 65 °C for 8 h and then cooled down to ambient temperature. The precipitation was filtered and dried at 80 °C for 2 h and calcined in a furnace at 550 °C for 4 h. The successfully obtained green ZnO samples with *Z. mays*, *A. heterophyllus* and *P. granatum* extracts were named as Z-ZnO, A-ZnO and P-ZnO, respectively (Figure 9).

For the characterization of ZnO NPs, different techniques were used: XRD, FTIR, FE-SEM, EDX, TEM, XPS, BET and UV-Vis spectrometry [52]. XRD results confirmed the hexagonal ZnO wurtzite phase, with the sizes of 28, 55 and 25 nm for the Z-ZnO, A-ZnO and P-ZnO. The FTIR spectrum also proved the presence of the three newly synthesized ZnO NPs. Using the FE-SEM technique, the morphologies of the nanostructures were also determined. According to these results, it can be seen that the Z-ZnO had a flower-like structure, A-ZnO had a cauliflower-like structure with a larger size, while P-ZnO was noticed as small nanoflowers with more branches at their center. The sizes of the newly synthesized NPs were in the range 650 nm−1.5 μm. The EDX analysis showed that the Zn:O atomic ratio was approximately 1:1. It also demonstrated that the Zn and O elements were evenly distributed throughout the product coverage area. The UV-Vis spectra also confirmed the presence of the ZnO. The BET analysis proved that the newly synthesized nanostructures had a higher surface area than the chemically synthesized ZnO, which is very important in the photocatalytic processes. In this study, the antibacterial effect of heterogeneous photocatalysis was tested. The findings showed that with Z-ZnO 93.2% of the microorganisms was removed, while in the systems with A-ZnO and P-ZnO, 85.7% and 99.2% of the bacteria were removed, respectively [52]. 

In another study, different metal oxides (including ZnO) were synthesized by green technique and combined with poly (methyl methacrylate) [53]. As a green component the leaf extract of *Sapindus mukorossi* was used. This was prepared by crushing the dried leaves and dissolving them in deionized water (10 mg/mL). For the green synthesis of the nanocomposites 0.56 g of different metal salt and 0.12 g of *S. mukorossi* was mixed with 25 mL of deionized water, while the components formed a colloidal suspension. After that, peroxydisulfate (0.12 g) was slowly added to this mixture and sonicated for 15 min by in-situ dropwise addition of 5 mL ammonium peroxydisulfate and poly(methyl methacrylate) (PMMA). Then, 5 mL of ammonium hydroxide was added and the mixture was refluxed at 150 °C for 4 h. In the end, the mixture was cooled, washed and dried at 50 °C for 12 h. For the characterization, different techniques were used: SEM, XRD, FTIR and BET [53]. The SEM results showed that all the MOs/PMMA nanocomposites were uniformly distributed, while ZnO-PMMA, Ni_2_O_3_-PMMA, CuO-PMMA were spherical-spirals containing MO as core bounded with PMMA matrix, while there also was a fine powder of Fe_3_O_4_-PMMA successfully synthesized. In order to determine the elemental composition of the newly synthesized nanocomposites, EDS analysis was applied and the finding showed that in CuO-PMMA C, S, O and Cu had weights of 50.80%, 0.98%, 39.45% and 8.77%, respectively. The ZnO-PMMA nanocomposite was found to contain 66.78%, 30.58% and 2.64% of C, O and Zn, respectively. In Fe_3_O_4_-PMMA, C, S, O and Fe were found at weights of 9.40%, 4.00%, 23.40% and 63.19%, respectively, whereas, in Ni_2_O_3_-PMMA, the C, O and Ni showed weights of 65.63%, 32.46% and 1.91%, respectively. XRD and FTIR findings also proved the successful formation of sustainably synthesized metal nanocomposites. The BET analysis showed that the surface areas of these new composites were higher than for pure oxides. Finally, the photocatalytic activity of these nanoparticles was also investigated in the degradation of MB using sunlight under different experimental conditions (initial pH, catalyst loading and concentration of MB). The best results were obtained with the lowest concentration of MB (2 mg/L), 80 mg of nanocomposites at neutral pH under sunlight. Under these optimal conditions, the synthesized nanocomposites showed different efficiency. Namely, ZnO-PMMA showed the highest photocatalytic removal capacity (99%) followed by Ni_2_O_3_-PMMA (98%) > CuO-PMMA (93%) > Fe_3_O_4_-PMMA (90%). Furthermore, the reusability of the catalysts was also examined. The findings showed that the nanocomposites were stable up to 10 times [53]. 

The curry leaf extract with coconut water was also investigated in the green synthesis of ZnO NPs by Satheshkumar et al. [54]. The leaves were initially washed and dried at ambient temperature. After that, they were ground to get a fine powder. Additionally, coconuts were smashed to collect the coconut water which was essential for the further extract preparation. The curry leaves (5 g) were stirred in 30 mL of coconut water at 50 °C for 2 h. Finally, the extract was filtered and stored at 10 °C for further use. For the synthesis of ZnO NPs, 20 mL of leaf extract was mixed with 30 mL of 0.1 M zinc nitrate solution under continuous stirring at 90 °C for 3–4 h. The originally green color of the solution turned into yellowish green which proved the formation of the nanoparticles. The formed NPs were filtrated, washed and dried which resulted in the formation of white color crystals. Finally, the crystals were transferred and kept in muffle furnace at 400 °C for 2 h. For the characterization, the following techniques were used: UV-Vis spectrometry, XRD, FTIR, FE-SEM and EDX [54]. The UV-Vis spectra proved the presence of the green synthesized ZnO NPs in the solutions. According to the XRD findings, it can be seen that the newly synthesized nanoparticles had hexagonal wurtzite structure with particle size 1.80, 1.62 and 1.88 nm with respect to 10, 15 and 20 mL concentrations of extract. FTIR results showed the characteristic functional groups present in coconut water, curry leaves’ extracts and ZnO NPs. The SEM images showed irregular spherical morphology with agglomeration, while the EDX analysis clearly exhibited the strong signal of Zn element and weak signal of O element. The weak signal of oxygen probably originated from the X-ray emission of macromolecules such as monoterpenes, tocopherols, lutein, proteins, sugars and vitamins present in the extracts. The photocatalytic activity of the green synthesized ZnO NPs was also examined in the degradation of MB under solar light irradiation. According to the results, it can be concluded that using these NPs as photocatalysts, 98.45% of MB was degraded after 60 min of irradiation [54]. 

*Stevia Rebaudiana* was also applied in the synthesis of ZnO for photocatalytic purposes [55]. For the extract preparation, 5 g of the *Stevia Rebaudiana* leaves was turned into a fine powder and dissolved in 100 mL of distilled water. This suspension was autoclaved for 30 min at 100 °C and under 103.4 kPa. Finally, the extract was filtered through Whatman No. 2 filter and stored in the refrigerator for further use. Afterwards, the synthesis of ZnO NPs was conducted using NaOH-assisted co-precipitation method. The presence of the Zn was ensured with 10 mL of 0.1 mM zinc nitrate hexahydrate solution (precursor), which was added to 100 mL of 10% *Stevia Rebaudiana* extract solution and stirred at ambient temperature. Next, the solution was heated at 50 °C and 40 mL of NaOH solution (0.2 M) was added dropwise into the reaction mixture, which resulted in the formation of cloudy precipitation. The reaction mixture was continuously stirred for 2 h at 50 °C and centrifuged at 4750 rpm × g for 10 min. The formed nanocomposites were then separated from the supernatant, rinsed with distilled water and separated by centrifugation. The purified nanopowder was dried at 60 °C for 4 h in an oven in order to turn remaining zinc hydroxide [Zn(OH)_2_] into ZnO. For the characterization the following techniques were used: UV-Vis spectrometry, FE-TEM, XRD and FTIR [55]. Based on UV-Vis results, the maximum absorbance peak was found to be at 366 nm which derives from the surface plasmon of oscillating electrons on SR-ZnO, resulting in the strong extinction of light. Additionally, the peak of the plant extract was absent which confirmed that the leaf extract did not affect the extinction spectrum of SR-ZnO. FE-TEM analysis showed that the newly synthesized NPs had flower-like structure, with four to six broad arrow petal-like structures. EDX spectra determined the highest optical peaks of zinc and oxygen, confirming the purity of SR-ZnO NPs. The XRD finding confirmed the hexagonal wurtzite structure of the ZnO NPs with the average size of 4.71 nm. Furthermore, the FTIR analysis proved the successful formation of ZnO NPs by this green method. On the other hand, the photocatalytic efficiency both of the pure leaf extract and the newly synthesized SR-ZnO NPs was also examined in the removal of MB. The findings showed that in the presence of the pure leaf extract 52% of MB was degraded, while in the presence of SR-ZnO 9% of MB was degraded as well, after 30 min of UV irradiation. The best results were conducted in the system with the combination of pure leaf extract and the newly synthesized NPs, where around 76% of MB was degraded after 30 min of UV irradiation. Increasing the reaction time to 60 min did not significantly improve sole photocatalytic activity of leaf extract and a mixture of SR-ZnO and leaf extract [55]. 

Another plant extract was investigated by the authors Tanase et al. [56] for the sustainable synthesis of ZnO for photocatalytic purposes. Firstly, *Saponaria officinalis* was prepared for the synthesis. The shredded roots of the plant were extracted in a Soxhlet apparatus for 1 h at 80 °C using 10 mL of the ethanol:water (1:1, *v/v*) mixture per gram of plant material. After that, the extract was filtrated through syringe filter (Minisart^®^ 0.8 nm) and stored in refrigerator for further use. There were four different ZnO NPs synthesized. Firstly, ZnO_1 was prepared dissolving 1.2 g of Zn(NO_3_)_2_·6H_2_O in 32 mL deionized water to which NaOH solution was added (composed of 1.6 g of sodium hydroxide dissolved 16 mL of deionized water). The mixture was stirred for 5 min and added to the microwave reactor. The second ZnO sample (ZnO_2) was prepared similarly, except in this case a cetyltrimethylammonium bromide solution (CTABr) was also added to the reaction mixture. Thirdly, ZnO_3 was prepared in the same way as ZnO_2, but for this synthesis 16 mL of alcoholic extract of *Saponaria Officinalis* and 16 mL of deionized water were added. Finally, ZnO_4 was created by the same procedure as for ZnO_1 with additional 16 mL of alcoholic extract of *Saponaria officinalis* and 16 mL of deionized water. Each of these samples were transferred into microwave vials and rapidly heated at 150 °C for 5 min without stirring. After, when the samples cooled down, they were washed with distilled water and dried at 90 °C for 2 h. For the characterization of the newly synthesized NPs, various techniques were applied: UV-Vis spectrometry, XRD, dynamic light scattering (DLS), laser Doppler velocimetry (LDV), SEM, EDX and XPS [56]. The ZnO formation was proved by the UV-Vis spectra, since a strong absorption peak appeared below 400 nm which is characteristic for the ZnO NPs. The XRD analysis confirmed the hexagonal wurtzite phase of the newly synthesized particles. Using SEM techniques determined the presence of various synthetic and natural structuring agents. Furthermore, flower-like and sword-like NPs were also observed in the SEM images. In addition, EDX spectra proved the presence of Zn and O in all the synthesized samples. The DLS results showed that the size of the newly synthesized ZnO NPs was between 42–5500 nm depending on the reaction conditions. The photocatalytic activity of these green synthesized nanocomposites was also investigated in the degradation of MB under visible light. ZnO_1 sample showed the highest efficiency, when 42% of MB was removed after 40 min of irradiation, while with the samples ZnO_2, ZnO_3 and ZnO_4, 33%, 21% and 15% of MB was degraded, respectively [56]. 

Muthukumar et al. [57] investigated the possible use of green synthesized ZnO NPs in the removal of toxic naphthalene from aqueous environment. The preparation of *Amaranthus dubius* leaf extract was done according to Harshiny et al. [58]. Namely, the freshly collected leaves of *Amaranthus dubius* were washed using deionized water and cut into small pieces. Twenty grams of the leaves were heated in 100 mL water at 50 °C for 45 min. After that, the colloid suspension was filtrated using Whatman No. 1 filter and the leaf extract was stored at 4 °C for further use. Subsequently, this extract was drop-by-drop added into the Zn(NO_3_)_2_ solution with continuous stirring at 37 °C for 30 min. The yellow precipitate was filtered through Whatman filter paper. Finally, the filtrate was dried in an oven at 90 °C for 5 h and stored in desiccators. Fe-doped ZnO was also prepared. In this synthesis, Fe-ZnO NPs, Zn(NO_3_)_2_ and ferric chloride were used as respective precursors for zinc and iron. They were mixed in appropriate proportions and the leaf extract (pH 9) was added drop by drop into the mixture and stirred at 37 °C for 30 min. As a result, a reddish yellow precipitate was formed which was filtrated, washed and dried at 90 °C for 5 h. The characterization was done using UV-Vis spectrometry, XRD, FTIR, SEM, EDS, electron paramagnetic resonance spectrometers (EMX-EPR) and XPS techniques [57]. The XRD analysis showed a cubic phase structure which is characteristic for ZnO. Moreover, the XRD peaks’ intensity was decreased with increasing the concentration of Fe^3+^ ions which resulted in defects in the ZnO crystal structure. The FTIR results confirmed the interactions of leaf extract on the NPs surface. The UV-Vis analysis additionally proved the successful synthesis of ZnO and Fe-ZnO nanoparticles. The photocatalytic activity of the newly synthesized NPs was tested in the removal of naphthalene under UV irradiation. The experiments were conducted in the presence of ZnO and Fe-ZnO NPs. The results showed that 63.5% and 71.7% of naphthalene was removed after 4 h of UV irradiation. Furthermore, UV-NIR spectra shown that Fe-doping significantly enhanced the light absorption capacity of ZnO in the visible region and also decreased bandgap from 3.2 to 2.91 eV [57].

Noukelag et al. [59] investigated the possible industrial dye removal using green synthesized Ag doped ZnO, as photocatalyst. For the biosynthesis *Rosemary leaves* were used. Mass of 2 g of the leaves was dissolved in 100 mL of boiled water at 80 °C for 2 h and the solution was filtrated twice. Then, 50 mg of AgNO_3_ and 450 mg of ZnCl_2_ were mixed with 100 mL of the extract and stirred at 60 °C for 1 h. After making the mixture uniform, 2 g of NaOH was added in order to set the pH to 10.58. The dark green solution was dried at 100 °C for 4 h. Finally, the powder was calcined at 500 °C for 2 h until the formation of gray Ag-ZnO NPs. Various techniques were used for characterization: FE-SEM, XRD, EDS, FTIR and UV-Vis-NIR [59]. The FE-SEM images showed a quasi-hexagonal structure of the NPs with high degree of agglomeration with average particle size of 28.946 ± 0.002 nm. In addition, the EDS findings confirmed the presence of Ag (3.94%), Zn (41.01%) and O (30.52%). The XRD analysis also proved the presence of Ag and ZnO. The size of the NPs was found to be in the range of 7.450–38.611 nm. The FTIR analysis confirmed the presence both of the Ag-ZnO NPs and the phenolic compounds, these proved the successful biosynthesis. The UV-Vis-NIR analysis showed that the bandgap of the newly synthesized NPs were decreased compared to the pure ZnO (i.e., the bandgap of the NPs was 3.27 eV, while of the pure ZnO it was 3.37 eV). The photocatalytic activity of the green synthesized NPs was also investigated in the photodegradation of textile effluent (TE) under visible light. The results showed that 63% of TE was removed after 100 min of visible irradiation, while in the absence of photocatalyst only 0.21% of TE was degraded [59]. 

The possible use of green synthesized chitosan/ZnO nanocomposite for antibacterial and photocatalytic activity was investigated by Preethi et al. [60]. In this synthesis, the leaf extract of *Solanum lycopersicum* was applied. Firstly, the leaves were washed, dried and cut into small pieces, after which they were ground using a kitchen blender. After that, 10 g of the leaves was extracted with 200 mL of ultrapure water using a Soxhlet extractor. The extract was stored at 4 °C for the experiments of green synthesis. In order to prepare the CS/ZnO nanocomposites, 50 mL of 0.5 M zinc sulfate was mixed with 25 mL of the prepared extract and stirred at 60 °C for 30 min. Then, 25 mL of chitosan solution was added to the reaction system and stirred again at 60 °C for 2 h. Finally, the precipitate was centrifuged at 6000 rpm for 15 min and dried at 80 °C for 24 h. The techniques which were used for the characterization are the following: UV-Vis spectrometry, XRD, SEM, TEM, EDS and FTIR [60]. The UV-Vis spectra of the newly synthesized CS/ZnO showed an absorbance peak at 360 nm which is characteristic of ZnO particles. The XRD spectrum confirmed the wurtzite structure with hexagonal phase of ZnO. The average crystal size of the new nanocomposite was 33 nm. Based on the SEM images it can be seen that the CS/ZnO particles were agglomerated with an average size of 21–47 nm. TEM images also showed regular spherical shaped structures with an average size of 25 to 70 nm. In synthesized nanocomposite, the EDS spectrum confirmed the existence of zinc (59%), oxygen (33%), carbon (6%) and nitrogen (2%). The Zn and O confirmed the successful formation of ZnO, while the presence of N proved the presence of CS, as well. The FTIR analysis additionally proved the formation of CS/ZnO nanocomposites. In order to determine the photocatalytic activity of CS/ZnO nanocomposites, experiments of photodegradation were conducted with CG dye after 300 min of sunlight irradiation. The obtained results showed that 80% of CG was removed after 300 min of sunlight irradiation [60].

**Table 3:** Basic information about the discussed methods of green synthesis and the main characteristics of the synthesized NPs.

Type of Catalyst	Applied Plant Extract in Experiments	Method of Synthesis	Size of the Newly Synthesized Particles	Structure of the Newly Synthesized Particles	Type of Pollutant in the Photocatalytic Experiments	Applied Irradiation	Efficiency of the Photocatalytic Degradation (%)	Reaction Rate Constant	Study
TiO_2_	Leaf extract of *Azadirachta indica*	Plant-mediated synthesis	Average crystal size in the range of 12.7–16.8 nm	Mesoporous structure of TiO_2_	Rhodamine 6 G	UV irradiation	64% after 57 min of irradiation	0.0321 min^−1^	[29]
TiO_2_	*Aloe Vera* gel from the plant leaf	Hydrothermal synthesis	Size of pure TiO_2_ 57 nm, while the Ag@TiO_2_ 38 nm	Combination of anatase and rutile phase	Picric acid	Visible irradiation	After 50 min of irradiation a decent amount of PA was removed	Not mentioned	[33]
TiO_2_	Leaf extract of *Cinnamomum camphora*	Synthesis under ambient conditions	12.6 ± 1.7 nm	Spherical shape and anatase phase of the Au-Ag/TiO_2_	methyl orange (MO), rhodamine B and methylene blue	UV-Vis irradiation (Xe lamp)	89.4% of MO after 60 min of irradiation; Complete degradation in the case of mixture dyes after 60 min of irradiation	0.0356 min^−1^ in the case of MO degradation; For the mixture the constant was not mentioned	[34]
TiO_2_	Leaf extract of *Deinbollia pinnata*	Sol–gel method	Average crystal size in the range of 19–21 nm	Aggregated, semi-spherical shape with anatase phase	Methyl orange	UV irradiation	97.53% after 150 min of irradiation	Not mentioned	[35]
TiO_2_	Leaf extract of *Euphorbia hirta*	Plant-mediated synthesis	Avarage crystal size in the range of 20–50 nm	Spherical shape and cubic phase of TiO_2_	Methylene blue (MB), MO, alizarin red (AR) and crystal violet (CV)	Direct sunlight	86.8% (CV); 81.3% (AR); 77.5% (MO) after 6 h of irradiation	Not mentioned	[36].
ZnO	Leaf extract of *Syzygium Cumini*	Not mentioned	11.35 nm	Agglomerated, well-crystallized hexagonal wurtzite structure	Methylene blue	Sunlight irradiation	91.4% after 180 min of irradiation	Not mentioned	[37]
ZnO	Pullulan, product of *Aureobasidium pullulans* fungus	Precipitation method	Average particle size 110.86 nm	Flower-like strucutre	Methyl orange	UV irradiation	97% after 300 min of irradiation	Not mentioned	[38,39]
ZnO	Leaf extract of *Cinnamomum tamala*	Plant-mediated synthesis	Average particle size 35 nm	Hexagonal wurtzite crystallite structure	Methylene blue	Direct sunlight	98.07% after 90 min of irradiation	Not mentioned	[40]
ZnO	Plant extract of *Gynostemma pentaphyllum*	Co-precipitation method	35.41 nm	Hexagonal structure of crystalline nanoparticles	Malachite green	UV irradiation	89% after 180 min of irradiation	Not mentioned	[41]
ZnO	Peel extract of *Cavendish* bananas	Plant-mediated synthesis	15.3 nm	Triangular and spherical shaped particles with hexagonal wurtzite structure	BB9 organic dye; Crystal violet (CV) and Congo red (CR)	UV-Vis irradiation (xenon lamp)	100% of BB9 after 90 min of irradiation; 97.79% of CV and 81.70% of CR after 420 min of irradiation	0.5254 h^−1^ for CV and 0.2837 h^−1^ for CR	[42]
ZnO	Leaf extract of *Alchornea laxiflora*	Plant-mediated synthesis	29–38 nm, depending on the volume of leaf extract	Quasi-hexagonal shape with hexagonal crystallographic phase	Congo red	Direct sunlight	87% after 60 min of irradiation	0.0401 min^−1^	[43]
ZnO	Peel extract of banana	Plant-mediated synthesis	18.86–20.72 depending on the type of banana	Nanocrystalline ZnO	Not mentioned	Not mentioned	Believed to be effective in the photodegradation	Not mentioned	[46]
ZnO	Jujube fruit extract	Plant-mediated synthesis	19 nm	Highly spherical shape with hexagonal wurtzite structure	Methylene blue (MB) and Eriochrome black-T (ECBT)	Direct sunlight	85% of both dyes after 300 min of irradiation	0.0087 min^−1^ for MB and 0.0067 min^−1^ for ECBT	[47]
ZnO	Leaf extract of *Prunus cerasifera*	Plant-mediated synthesis	Average crystal size 12 nm	Aggregated spheroidal shape with wurtzite hexagonal phase	Bromocresol green (BG), Bromophenol Blue (BB), Methyl red (MR) and Methyl blue (MB)	Direct sunlight	93.12% of BG; 90.54% of BB; 88.49% of MR and 76.76% of MB after 10 min of irradiation	Not mentioned	[48]
ZnO	Leaf extract of *Becium grandiflorum*	Biological approach	Average crystal size of 20 nm	Hexagonal wurtzite structure	Methylene blue	UV irradiation	69% after 200 min of irradiation	0.0019 min^−1^	[49]
ZnO	Root extract of *Codonopsis lanceolata*	Modified co-precipitation method	500 nm	Spherical, flower-like shape with hexagonal wurtzite structure of ZnO	Methylene blue	UV irradiation	90.3% after 40 min of irradiation	0.057 min^−1^	[50]
ZnO	Leef extract of *Peltophorum pterocarpum*	Plant-mediated synthesis	11.64 nm	Flowershaped particles with hexagonal wurtzite phase of ZnO	Methylene blue	Sunlight irradiation	95% after 120 min of irradiation	0.021 min^−1^	[51]
ZnO	Husk extract of *Zea mays* (Z-ZnO) and peel extract of *Artocarpus heterophyllus* (A-ZnO) and *Punica granatum* (P-ZnO)	Co-precipitation method under low temperature	28 (Z-ZnO), 55 (A-ZnO) and 25 (P-ZnO) nm	Z-ZnO flower-like; A-ZnO cauliflower-like and P-ZnO small nanoflower structure with hexagonal ZnO wurtzite phase	Antibacterial activity	Visible light irradiation	93.2% (Z-ZnO), 85.7% (A-ZnO) and 99.2% (P-ZnO) after 180 min of irradiation	0.0130 (Z-ZnO), 0.0091 (A-ZnO) and 0.0280 (p-ZnO) min^−1^	[52]
ZnO	Leaf extract of *Sapindus mukorossi*	Plant-mediated synthesis	10–1000 nm	Spherical-spiral shape	Methylene blue	Sunlight irradiation	99% (ZnO-PMMA); 98% (N_i2_O_3_-PMMA); 93% (CuO-PMMA); 90% (Fe_3_O_4_-PMMA) after 130 min of irradiation	0.1349 (ZnO-PMMA); 0.1321 (N_i2_O_3_-PMMA); 0.1263 (CuO-PMMA); 0.1231 (Fe_3_O_4_-PMMA) min^−1^	[53]
ZnO	Leaf extract of curry with coconut water	Plant-mediated synthesis	1.80, 1.62 and 1.88 nm with respect to 10-, 15- and 20-mL concentration of extract	Agglomerated, irregular spherical shape	Methylene blue	Sunlight irradiation	98.45% after 60 min of irradiation	0.0579 min^−1^	[54]
ZnO	Leaf extract of *Stevia rebaudiana*	Co-precipitation method	Average crystallite size 4.71 nm	Agglomerated flower-like shape with hexagonal wurtzite structure of the ZnO	Methylene blue	UV irradiation	76% after 30 min of irradiation	Not mentioned	[55]
ZnO	Root extract of *Saponaria officinalis*	Precipitation method	42–5500 nm	Sowrd-like shapes with hexagonal wurtzite phase of ZnO	Methylene blue	Visible light irradiation	15–42% depending on the applied catalyst, after 40 min of irradiation	Lower than the used reference value (i.e. lower than 0.0344 min^−1^)	[56]
ZnO	Leaf extract of *Amaranthus dubius*	Plant-mediated synthesis	82–250 nm for ZnO and 71–280 nm for 1% Fe-ZnO	Spherical cubic phase	Naphthalene	UV irradiation	63.5% (ZnO) and 71.7% (Fe-ZnO) after 240 min of irradiation	0.0045 (ZnO) and 0.0054 (Fe-ZnO) min^−1^	[57]
ZnO	Leaf extract of Rosemary	Plant-mediated synthesis	Average crystalline size 28.946 ± 0.002 nm	Quasi-hexagonal structure with high degree of agglomeration	Textile effluent	Visible light irradiation	63% after 100 min of irradiation	0.0111 s^−1^	[59]
ZnO	Leaf extract of *Solanum lycopersicum*	Plant-mediated synthesis	Average crystalline size 33 nm	Agglomerated spherical shape with hexagonal wurtzite structure of ZnO	Congo red	Sunlight irradiation	80% after 300 min of irradiation	Not mentioned	[60]

## 3. Green Synthesis of Special Nanomaterials Based on TiO_2_ and ZnO

Special nanomaterials are unique, as they do not exist in nature and are truly “man-made” materials with different and commonly difficult synthetic pathways. These compounds possess various, extraordinary physical and chemical properties. Some of them are: carbon fullerenes and nanotubes, ordered mesoporous materials, organic inorganic hybrids, intercalation compounds, and oxide-metal core-shell structures, etc. These compounds can be successfully used in different fields such as: optics, electronics, sensors, ionics, energy conversion and storage, mechanics, membranes, protective coatings, catalysis, etc. Even though that these compounds are intensively investigated and their application is very popular, their synthetic routes require application of different chemicals, which makes them harmful for the environment. Hence, different green approaches should be developed in order to reduce their footprints in the nature. In the following, several studies about the green synthesized special nanomaterials for photocatalytic purposes will be discussed [61,62]. 

Aragaw et al. [63] investigated the possible sustainable synthesis of the p-Co_3_O_4_/n-ZnO composite as catalyst and its application in the photodegradation of MB under visible light irradiation. *Eichhornia Crassipes* was firstly collected then washed with tap and distilled water, after which the plant was dried at ambient temperature. Afterwards, 15 g of the plant powder was dissolved in 430 mL of water and stirred at 50 °C for 1 h. Finally, the mixture was filtrated and stored for further use. For the preparation of p-Co_3_O_4_/n-ZnO NPs a co-precipitation method was applied. A volume of 40 mL of Zn(NO_3_)_2_·6H_2_O (0.1 M) and 40 mL of Co(NO_3_)_2_·6H_2_O (0.1 M) were mixed with 40 mL of plant extract and stirred for 2 h. Additionally, 2 M of NaOH was drop-by-drop added to the reaction mixture in order to form hydroxide colloidal solution. After that, the solution was centrifuged and washed with distilled water and ethanol. The precipitate was dried at 60 °C and calcined at 500 °C for 2 h. For the characterization, the following techniques were applied: XRD, SEM, EDX, FTIR and UV-Vis spectrometry [63]. XRD analysis proved the hexagonal structure of ZnO as well as the cubic phase of Co_3_O_4_. The average crystal sizes of ZnO and p-Co_3_O_4_/n-ZnO were 24.62 and 16.68 nm, respectively. The peaks in the FTIR spectra proved the presence of Co-O and Zn-O bonds; hence, they confirmed that the p-Co_3_O_4_/n-ZnO NPs were synthesized successfully. The SEM and EDX analysis also proved the presence of the newly synthesized NPs. The findings of the photocatalytic degradation showed that in the system with the green synthesized NPs, 95.5% of the MB dye was degraded after 60 min of visible light irradiation. Lower degradation was found in the systems with pure ZnO and Co_3_O_4_ (i.e., 72% and 79% of the MB were degraded after 60 min of irradiation, respectively). According to these results, it can be concluded that the *Eichhornia Crassipes* plant extract mediated the p-Co_3_O_4_/n-ZnO composite, and could be successfully applied for water purifications [63]. 

**Figure 10:**
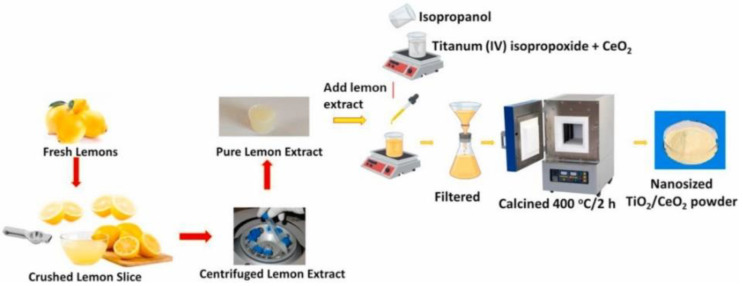
Green preparation of TiO_2_–CeO_2_ nanocomposites. Reprinted with permission from Ref. [64], Copyright 2021 Elsevier.

The possible photocatalytic use of the combined TiO_2_ and CeO_2_, which was sustainably synthesized in the presence of lemon extract, was examined by Gnanasekaran et al. [64]. Firstly, the lemon extract was prepared. The fresh lemons were crushed, filtered and centrifuged to remove the present impurities. At the beginning, the pure TiO_2_ and CeO_2_ nanopowders were synthesized. For the TiO_2_ synthesis the sol–gel method was applied. Firstly, 3 mL of TTIP was mixed with 100 mL of isopropanol and the reaction mixture was stirred for more than 1 h. After that, 10 mL of lemon extract was added to this solution and stirred for 24 h. The yellowish-white powder was left at ambient temperature and transferred to the mortar for grinding. Finally, the powder was calcined at 350 °C for 2 h which resulted in the formation of white powder. Secondly, the pure CeO_2_ was also synthesized using this green technique. In this case, the precipitation method was applied. For this purpose, 0.05 M cerium(II) chloride (metal precursor) was dissolved in 200 mL of distilled water to which 10 mL of the lemon extract was added, as well as sodium hydroxide (0.5 M). This resulted in the formation of a precipitate (at pH level 7 to 8). After removing the impurities, the precipitate was dried for 1 h and calcined at 350 °C for 2 h. Nevertheless, TiO_2_–CeO_2_ composite materials were also green synthesized, namely by combining the sol–gel and precipitation techniques. The exact ratios of TTIP, CeO_2_ obtained from the precipitation method and isopropanol (1:0.1:5) were mixed and stirred for 1 h. After that, 10 mL of the lemon extract was added to the mixture until the solution turned into a gel. Finally, the gel was dried and ground into powder and calcined at 350 °C for 2 h (Figure 10).

For the characterization of the newly synthesized nanocomposites, different techniques were used: XRD, TEM, EDX, FTIR, BET and UV-Vis spectrometry [64]. The XRD results showed that the synthesized TiO_2_ (with and without lemon extract) had a tetragonal structure and anatase phase without any impurities and with the sizes of 19.5 and 15.3 nm, respectively. In the case of CeO_2_, XRD determined the presence of a cubic structure with the size of 10.1 nm. Furthermore, in the case of the green synthesized nanocomposite, the combined tetragonal and cubic structures were detected, with crystal size of 9.4 nm. The EDX and TEM analyses clearly proved the presence of the pure TiO_2_, CeO_2_ as well as the newly synthesized nanocomposite. Additionally, the elemental mapping proved the presence of Ti, Ce, and O elements. The FTIR results additionally confirmed the successful synthesis of the nanocomposites. The photocatalytic activity of the newly synthesized TiO_2_, CeO_2_ as well as the TiO_2_–CeO_2_ composite was investigated in the photodegradation of 2,4-dichlorophenol (2,4-DCP) under simulated sunlight irradiation. According to these results, the highest efficiency was observed in the case of the nanocomposite, when 49% of 2,4-DCP was removed after 6 h of visible light irradiation [64]. 

In the study of Rachna et al. [65], a green synthesized ZnO@FeHCF nanocomposite was investigated in the degradation of bisphenol A (BPA) and nonylphenol (NP). For the biosynthesis the extract of *Azadirachta indica* leaves was used. The extract was prepared collecting, washing and grinding the leaves. Five grams of the leaves was dissolved in 10 mL of distilled water. The solution was stored at 4 °C for further use. The synthesis of nanocomposites was conducted in two steps. Firstly, the ZnO nanosheets were prepared. The leaf extract (2 mL) was mixed with 0.05 M Zn(NO_3_)_2_ and kept for 1 h stirring. Then, using NaOH, the pH was set to 9 which resulted in the precipitation of bleached particles. The product was filtered, washed and dried at 300 °C for 4 h. In the second step, the ZnO@FeHCF nanocomposite was produced. The encapsulation of FeHCF was carried out by sonicating the ZnO NPs in small portions of 100 mL ethanol, and stirred for 30 min. Then, 100 mL of 0.1 M Fe(NO_3_)_2_, K_4_[Fe(CN)_6_] and 4 mL of leaf extract were added to the above mentioned suspension and stirred for 2 h. As a result, a blue-colored precipitate has appeared which was filtrated, washed and dried at 70 °C. For the characterization of the newly synthesized nanocomposite, the following techniques were applied: XRD, FE-SEM, FTIR, DRS and BET analysis [65]. The XRD data showed a hexagonal wurtzite-like structure in the case of ZnO and a cubic lattice for FeHCF. Additionally, the sharp peaks proved the high crystallinity and purity. Incorporation of the parent materials in the nanocomposite was determined by the slight shift in the XRD parameters. The FE-SEM images showed a flake-like geometry in the case of ZnO@FeHCF nanocomposites with irregular surface comprising ZnO nanoflowers within nanorange. The EDS proved the presence of O and ZnO in the nanocomposite which confirmed the coupling of FeHCF nanoparticles by ZnO. The FTIR spectra additionally proved the successful synthesis. On the other hand, the DRS findings showed that coupling ZnO with FeHCF decreases the bandgap energy from 3.3 eV (ZnO) and 1.2 eV (FeHCF) to 1.1 eV (ZnO@FeHCF). This resulted in higher activity under visible irradiation. Furthermore, the BET analysis showed a higher surface area in the case of ZnO@FeHCF (80 m^2^/g), while in the case of ZnO and FeHCF, the areas were 12 and 69 m^2^/g, respectively [65]. The photocatalytic activity of the green synthesized nanocomposites was also investigated in the photodegradation of endocrine phenols under simulated sunlight and different experimental conditions. The optimized photocatalytic conditions were the following: A total of 2 mg/mL catalyst loading, with 1.6 × 10^−3^ substrate concentration mg/L and at neutral pH. The highest efficiency was reached in the case of ZnO@FeHCF, where 94% and 91% of BPA and NP were removed after 24 h of irradiation, respectively. The FeHCF and ZnO showed a slightly less photocatalytic efficiency, where 78% of BPA, 73% of NP; and 71% of BPA and 67% of NP were removed, respectively, after 24 h of irradiation. Furthermore, the catalyst reusability experiments showed that they were stable at least 10 cycles of photodegradation [65]. 

ZnO nanorods synthesized using sustainable pathways were also investigated for photocatalytic activity by Vidya et al. [66]. For the green synthesis, *Calotropis gigantea* leaf extract was used, which was prepared as follows: Firstly, the collected leaves were washed and dried in sun; after that 50 g of them was cut into small pieces and mixed with 500 mL of distilled water. This mixture was boiled until the water content was reduced up to 50% of its initial volume, which resulted in a brown extract; this extract was cooled to ambient temperature and stored at low temperature for further use. For the synthesis of ZnO nanorods, 100 mL of the leaf extract was firstly boiled and stirred at 60–80 °C, and when the extract reached 80 °C, 5 g of zinc nitrate hexahydrate (Zn(NO)_3_·6(H_2_O)) was added and boiled until a deep yellow paste appeared. Finally, this paste was then calcined at 400 °C for 1 h. For the characterization, various techniques were used, such as XRD, SEM, TEM, EDS, FTIR and UV-Vis spectrometry [66]. XRD results proved the wurtzite structure of ZnO as well as the presence of some impurities. The SEM images proved the successful formation of hexagonal nanorods (ZNRs). These ZNRs had length between 20 and 200 nm, while their diameters were in a range of 20–80 nm with width between 20 and 50 nm. The TEM and FTIR results additionally confirmed the formation of ZnO nanorods. Furthermore, EDS spectra proved that there were not impurities in the synthesized ZNRs. In addition, the findings also determined the presence of zinc and oxygen in concentrations of 85.32% and 14.68%, respectively. The photocatalytic degradation experiments were conducted with titan yellow dye (TY) under different experimental conditions with UV irradiation. The effect of different factors was examined, and according to the results it can be concluded that the initial dye concentration had strong influence on the efficiency of TY degradation. The highest removal percentage (more than 90%) was reached with 10 ppm TY, while with 20, 30 and 40 ppm the removal was 60%, 60% and 50%, respectively. The effect of catalyst loading was also investigated with 20, 40, 60 and 80 mg of ZNRs in 250 mL of 20 ppm dye concentration. The findings showed that the efficiency increased up to 60 mg; however, with higher catalyst loading the efficiency decreased. The influence of initial pH was also questioned. The results showed that the percentage of TY degradation increased as the pH increased from 6 to 8, while at pH 10 the efficiency decreased. In conclusion, the obtained results showed that, under optimal conditions (10 ppm TY, 60 mg catalyst loading and initial pH 8), more than 95% of TY can be removed from the investigated systems. Hence, the green synthesized ZNRs can be potential catalysts for water remediation [66]. 

**Figure 11:**
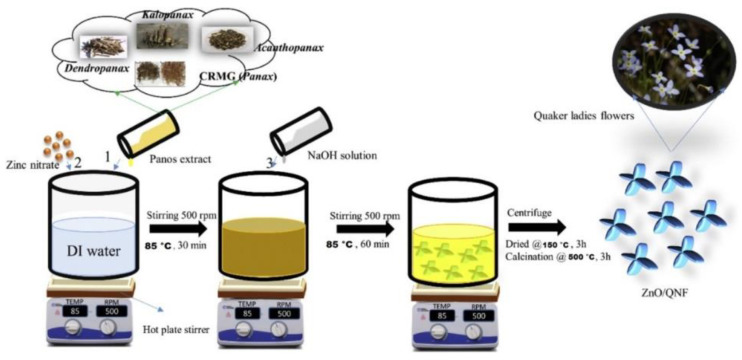
Scheme of the green synthesis of ZnO nanoflowers. Reprinted with permission from Ref. [67], Copyright 2021 Elsevier.

Kaliraj et al. [67] investigated the green synthesis of ZnO nano-flowers (ZnO/QNF) for photocatalytic degradation of dye. In this green synthesis, different plants took place. Namely, roots of *Panax ginseng* and stem portions of *Acanthopanax senticosus*, *Kalopanax septemlobus* and *Dendropanax morbifera*. Firstly, the roots and barks were rinsed with distilled water and cut into small pieces in order to get fine powder. Then, they were mixed with water, which was added in 1:8 ratio (raw material:water) and the extraction was conducted at 85 °C for 8 h. After the extraction, the mixture was filtrated and the extracts were concentrated under reduced pressure of 800–850 mm/Hg at 60 °C. Finally, the panos extract for further experiments was prepared by mixing 25% of each plant extract. The synthesis of ZnO nano-flowers was conducted using the co-precipitation method. Zinc nitrate was used as a source of Zn in the synthesis of NPs, while the panos extract acted as a reducing agent. Firstly, a mixture was prepared by adding 1% of panos extract to 99 mL of distilled water. To this solution, 10 mL of 0.15 M zinc nitrate was added dropwise. The mixture was heated at 85 °C with constant stirring for 30 min. After that, 25 mL of 2 M NaOH was added drop-by-drop to the stirred mixture, which was followed by rapid stirring at 500 rpm for 2 h. Then, the mixture was kept at ambient conditions overnight. Afterwards, the solution was centrifuged at 8000 rpm for 10 min at ambient temperature. Finally, the pellets were washed with distilled water and dried at 150 °C for 3 h and calcined at 500 °C for 3 h (Figure 11).

Various techniques were applied for the characterization: XRD, XPS, FTIR, TEM and SAED [67]. The XRD results confirmed the wurtzite structure of ZnO. The TEM analysis proved the four-way leafy flower-like structure with the size of 480 nm (pedals of 240 nm in length and 120 nm in width), while the SAED determined the fringe length of about 0.21 nm. In the FTIR spectra the absence of Zn-(H_2_O) peaks and presence of Zn-O, Zn-OH (surface hydroxyl) proved the formation the new ZnO nano-flowers. Furthermore, the XPS findings showed a significant presence of Zn, O and C, which is an additional proof of the successful green synthesis of the new nanoparticles. The photocatalytic studies were conducted in the presence of MB, eosin Y (EY) and MG dyes under UV irradiation. It was found that >99% of MB, EY and MG was removed after 80, 90 and 110 min of contact time with ZnO/QNF, respectively. Furthermore, at low dye concentration (5 mg/L), 100% removal efficiency was reached within 30, 35 and 40 min of irradiation for MB, EY and MG, respectively. The reusability of the newly synthesized catalyst was also examined. The results showed that the photocatalytic efficiency after five runs was only a slightly decreased in the degradation of all three dyes (Table 4) [67].

## 4. Conclusions

This review discussed different green approaches in the synthesis of TiO_2_ and ZnO nanoparticles, mostly based on leaf extracts, for photocatalytic purposes. Heterogeneous photocatalysis is a promising method for water purification. However, the generally used semiconductors have limitations, such as the possible recombination of photogenerated electron–hole pairs and the high bandgap energy. Thus, it is necessary to enhance their activity. Nanomaterials, thanks to their size in the nanoscales, have unique and extraordinary properties and they can be also used as photocatalysts. The application of green techniques is becoming popular method for synthesis of new, and improvement of already known, nanomaterials. This fact is confirmed with the growing number of publications about this topic. According to the discussed studies, it can be concluded that the green synthesized nanomaterials in many cases defeated the traditionally prepared TiO_2_ and ZnO. Among the examined studies, the most sustainable results were obtained in the presence of ZnO NPs synthesized in the presence of the leaf extract of curry with coconut water, when 98.45% of MB was degraded after 60 min of sunlight irradiation. Not only do these compounds commonly have various sizes in nanoscale, higher specific surface area available for reaction or adsorption, but they also have better absorbance properties in the visible region of electromagnetic radiation. This is a very important progress, since the natural sunlight is free, renewable and available for everyone. Meaning that, in this way, the photocatalysts could harvest photons from the natural irradiation source. Moreover, the plant extracts used for the sustainable synthesis can additionally improve the photocatalytic activity, because of the biomolecules and other natural compounds present in them. 

## 5. Challenges and Future Development 

As the world’s population is continuously growing, environmental pollution, especially water contamination, is becoming an emerging global issue. Hence, there is no doubt that the application of heterogeneous photocatalysis and nanomaterials are the future in water remediation techniques, as well as in many other fields. Unfortunately, there are some challenges which have to be overcome for the successful application of photocatalysis in the wastewater treatment plants. To begin with, the most commonly used semiconductors in photocatalysis possess different drawbacks. For instance, because of the wide bandgap of TiO_2_ and ZnO, the application of artificial UV radiation is necessary. In that regards, the whole process becomes more expensive and additional safety equipment has to be used to be protected from the UV light. Furthermore, there are many factors which affect the efficiency of photocatalysis. These factors are usually difficult to be optimized under conditions outside the laboratory. For the mentioned reasons, in the future scientists and engineers should put emphasis on the development of an efficient photocatalyst which can be activated under natural sunlight. Moreover, the whole process of heterogeneous photocatalysis should be simplified and made more user-friendly, less costly and globally available. The application of the nanotechnology and nanomaterials is undoubtedly one of the possible solutions for making heterogeneous photocatalysis more practical. However, the most commonly used methods for fabricating nanoparticles have some disadvantages, as well. Firstly, the generally applied techniques for nanomaterial synthesis are commonly difficult and require a decent amount of harmful chemicals, which additionally pollutes the environment. Secondly, the newly synthesized NPs can be aggregated/agglomerated which results in reduced catalytic activity. Hence, in the future the synthesis of nanomaterials should be exclusively based on the application of green techniques (e.g., application of plant extracts), as well as the agglomeration/aggregation should be avoided during and after the synthesis. The positive effect of the plant extracts in the synthesis of NPs is well proved in many researches. However, this field requires further investigation, since we are not entirely familiar with the effects of biomolecules both in the synthesis and in the photocatalytic processes. Furthermore, the classic extraction techniques require large amount of chemicals, so green extraction techniques also should be developed (e.g., microwave extraction). In this way, we could completely reduce our footprint in the environment, but also develop materials which could be used for improving the sanitary conditions all across the Earth. 

## Figures and Tables

**Table 1:** Basic properties of titanium dioxide. Adapted from Ref. [13].

Properties	Values
CAS number	13463-67-7
Molecular formula	TiO_2_
Molar mass	79.866 g/mol
Appearance	White powder
Odor	Odorless
Density	3.9 to 4.2 g/mL
Melting point	1860 °C (decomposes)
Boiling point	2500–3000 °C
Solubility in water	Less than 1 mg/mL (20 °C)
Band gap	3.2 eV (anatase); 3.02 eV (rutile)
Refractive index (nD)	2.554 (anatase); 2.583 (brookite); 4.17 (rutile)

**Table 2:** Basic properties of zinc oxide. Adapted from Ref. [18].

Properties	Values
CAS number	1314-13-2
Molecular formula	ZnO
Molar mass	81.408 g/mol
Appearance	White solid
Odor	Odorless
Density	5.606 g/mL
Melting point	1975 °C (decomposes)
Boiling point	2360 °C
Solubility in water	0.16 mg/100 mL (30 °C)
Band gap	3.3 eV (direct)
Refractive index (nD)	2.0041

**Table 4:** Summary of the green-inspired special nanomaterials.

Type of Catalyst	Applied Plant Extract in Experiments	Method of Synthesis	Size of the Newly Synthesized Particles	Structure of the Newly Synthesized Particles	Type of Pollutant in the Photocatalytic Experiments	Applied Irradiation	Efficiency of the Photocatalytic Degradation (%)	Reaction Rate Constant	Study
p-Co_3_O_4_/n-ZnO	Plant extract of Eichhornia Crassipes	Co-precipitation method	Average crystal size 16.68 nm	Clusters of close packed organization with hexagonal phase of ZnO and cubic phase of Co_3_O_4_.	Methylene blue	Simulated sunlight	95.5% of the MB, after 60 min of irradiation	0.028 min^−1^	[63]
TiO_2_–CeO_2_	Lemon extract	Combination of sol–gel and precipitation method	9.4 nm	Combined tetragonal and cubic structures	2,4-dichlorophenol (2,4 DCP)	Simulated sunlight	49% of 2,4-DCP was removed after 300 min of irradiation	Not mentioned	[64]
ZnO@FeHCF	Leaf extract of *Azadirachta indica*	Plant-mediated synthesis	Size within nanorange (100 nm)	Flakes like geometry; hexagonal wurtzite-like structure in the case of ZnO and cubic lattice for FeHCF	bisphenol A (BPA) and nonylphenol (NP)	Simulated sunlight	94% (BPA) and 91% (NP) after 24 h of irradiation	0.2797 h^−1^ (BPA) and 0.2663 h^−1^ (NP)	[65]
ZnO nanorods	Leaf extract of *Calotropis gigantea*	Plant-mediated synthesis	Length between 20 and 200 nm; diameter between 20 and 80 nm; and width between 20 and 50 nm	Nano-rods in hexagonal pattern	Titan yellow (TY)	UV irradiation	>95% (under optimal conditions) after 60 min of irradiation	Average rate found to be 35, 37 and 40 min^−1^	[66]
ZnO nanoflowers	Panos extract	Co-precipitation method	480 nm (pedals of 240 nm in length and 120 nm in width)	Four-way leafy flower-like structure with confirmed wurtzite phase of ZnO	Methylene blue (MB), eosin Y (EY) and malachite green (MG)	UV irradiation	>99% of MB, EY and MG was removed after 80 min, 90 min and 110 min of irradiation	Not mentioned	[67]

## Data Availability

Not applicable.
